# Integrative single-cell and bulk transcriptomes analyses reveals heterogeneity of serine-glycine-one-carbon metabolism with distinct prognoses and therapeutic vulnerabilities in HNSCC

**DOI:** 10.1038/s41368-024-00310-2

**Published:** 2024-06-17

**Authors:** Lixuan Wang, Rongchun Yang, Yue Kong, Jing Zhou, Yingyao Chen, Rui Li, Chuwen Chen, Xinran Tang, Xiaobing Chen, Juan Xia, Xijuan Chen, Bin Cheng, Xianyue Ren

**Affiliations:** 1 Hospital of Stomatology, Sun Yat-Sen University, Guangzhou, China; 2 Guangdong Provincial Key Laboratory of Stomatology, Guangzhou, China; 3 Guanghua School of Stomatology, Sun Yat-Sen University, Guangzhou, China; 4 Department of Radiation Oncology, Nanfang Hospital, Southern Medical University, Guangzhou, China

**Keywords:** Cancer metabolism, Head and neck cancer, Tumour biomarkers

## Abstract

Metabolic heterogeneity plays a central role in sustaining uncontrolled cancer cell proliferation and shaping the tumor microenvironment (TME), which significantly compromises the clinical outcomes and responses to therapy in head and neck squamous cell carcinoma (HNSCC) patients. This highlights the urgent need to delineate the intrinsic heterogeneity and biological roles of metabolic vulnerabilities to advance precision oncology. The metabolic heterogeneity of malignant cells was identified using single-cell RNA sequencing (scRNA-seq) profiles and validated through bulk transcriptomes. Serine–glycine-one-carbon (SGOC) metabolism was screened out to be responsible for the aggressive malignant properties and poor prognosis in HNSCC patients. A 4-SGOC gene prognostic signature, constructed by LASSO-COX regression analysis, demonstrated good predictive performance for overall survival and therapeutic responses. Patients in the low-risk group exhibited greater infiltration of exhausted CD8^+^ T cells, and demonstrated better clinical outcomes after receiving immunotherapy and chemotherapy. Conversely, high-risk patients exhibited characteristics of cold tumors, with enhanced IMPDH1-mediated purine biosynthesis, resulting in poor responses to current therapies. IMPDH1 emerged as a potential therapeutic metabolic target. Treatment with IMPDH inhibitors effectively suppressed HNSCC cell proliferation and metastasis and induced apoptosis in vitro and in vivo by triggering GTP-exhaustion nucleolar stress. Our findings underscore the metabolic vulnerabilities of HNSCC in facilitating accurate patient stratification and individualized precise metabolic-targeted treatment.

## Introduction

Cancers are characterized by uncontrolled cell proliferation and can thrive in a changing microenvironment by rewiring metabolic processes to provide nutrients and energy, activate oncogenic signaling pathways, and manage redox homeostasis.^1,2^ The serine-glycine-one-carbon (SGOC) metabolic network incorporates serine-glycine biosynthesis, folate and methionine cycles, and purine nucleotide biosynthesis in a positive feedback loop, which can satisfy many of cancer cells’ requirements.^3^ Moreover, the products and intermediates of SGOC metabolism can educate immune cells in the tumor microenvironment (TME), such as cyclic dinucleotides, which play critical roles in cancer immunotherapy. Increasing evidence demonstrates that highly proliferative cancer cells exhibit increased expressions of SGOC metabolic enzymes.^3–5^ SGOC metabolism may represent a vulnerability in highly SGOC-activated tumors, and SGOC metabolic enzymes may be potential therapeutic target genes for cancer treatment in future scenarios.^3^ Therefore, understanding the roles of SGOC metabolism in tumorigenesis and their relationship with anti-cancer therapy is of great significance.

The increase in the ribonucleic acid (RNA) to deoxyribonucleic acid (DNA) ratio in growing cells indicates an enhanced overall biosynthetic capacity during malignant transformation, which could result in the enlargement of nucleolar morphology, a phenomenon known as nucleolar hypertrophy.^6^ De novo synthesis of purine nucleotides rapidly incorporates into RNA in proliferating cells, which is the main reason for nucleolar hypertrophy.^7^ Inosine 5’-monophosphate dehydrogenase (IMPDH) catalyzes the oxidative conversion of inosine 5’-monophosphate (IMP) into xanthosine 5’-monophosphate (XMP) and controls the gateway to guanine nucleotides. It is the key enzyme of de novo purine synthesis.^8^ Upregulation of IMPDH, which induces guanine nucleotide accumulation, has been identified to be associated with malignant transformation in several cancers, such as glioblastoma and lung cancers. Thus, suppression of IMPDH-regulated de novo purine biosynthesis represents a promising therapeutic strategy. Currently, IMPDH inhibitors, including mycophenolic acid (MPA, CellCept®), mizoribine (Bredinin®), and ribavirin (Virazole® and Rebetol®), are widely used in the clinic as antivirals.^8^ However, the application of IMPDH inhibitors in anti-tumor therapy still need to be elucidated.

Head and neck squamous cell carcinoma (HNSCC) ranks as the 7th most common cancer type worldwide, characterized by high heterogeneities and mortality rates.^9,10^ While multi-modal therapeutic strategies have significantly improved over the past decades, including chemotherapy and immunotherapy, fewer than 60% of patients achieve the 5-year survival mark with chemotherapy, and less than 20% of patients with advanced disease respond to immune checkpoint blockade-based immunotherapies in HNSCC.^11^ This underscores the inadequacy of the existing prognostic predictive system and standard therapy for high-risk patients. Considering the central roles of metabolism in tumorigenesis and development, systematically characterizing intra- and inter-tumoral metabolic heterogeneity will aid in the search for individualized, precise treatment strategies.

Previously studies have established that HNSCC is a metabolic disorder disease primarily based on bulk transcriptomic profiles,^12^ which significantly obscure the heterogeneous cancer ecosystem and compromise the accuracy and clinical utility of metabolic-based biomarkers. Therefore, the aim of this study was to unveil an intrinsic malignant classification for HNSCC based on metabolic heterogeneity using scRNA-seq profiles, which enable precise characterization of cellular heterogeneity and plasticity in the complex cancer ecosystem. We explored the relationship between differentially expressed metabolic pathways and patients’ clinical outcomes from bulk RNA-seq data using scRNA-seq reference as prior information. A novel, robust 4-gene signature based on SGOC was developed to stratify HNSCC patients into high- and low-risk groups. The differences in the TME and response to therapies were analyzed. Furthermore, the anti-tumor efficacy of novel metabolic-targeted drugs was evaluated in vitro and in vivo. Overall, our work sheds novel light on metabolic heterogeneity and metabolic-targeted clinical strategies for HNSCC patients.

## Results

### SGOC upregulation represents a prominent metabolic feature in HNSCC

**Fig. 1:**
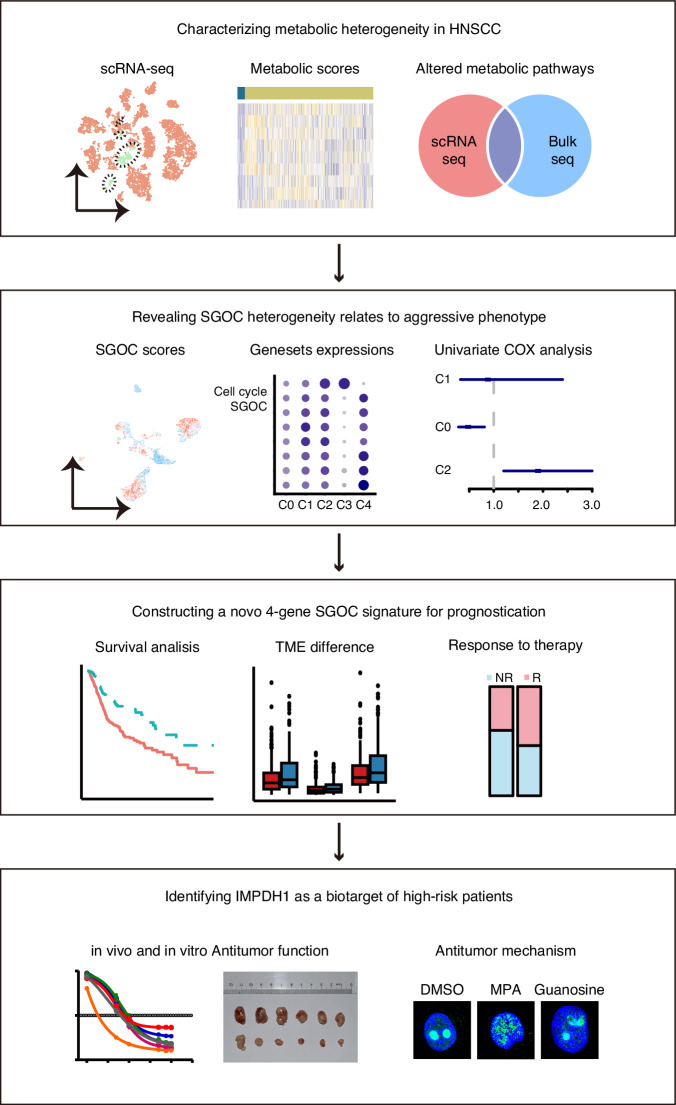
Overall design and analytic pipeline of this study

**Fig. 2:**
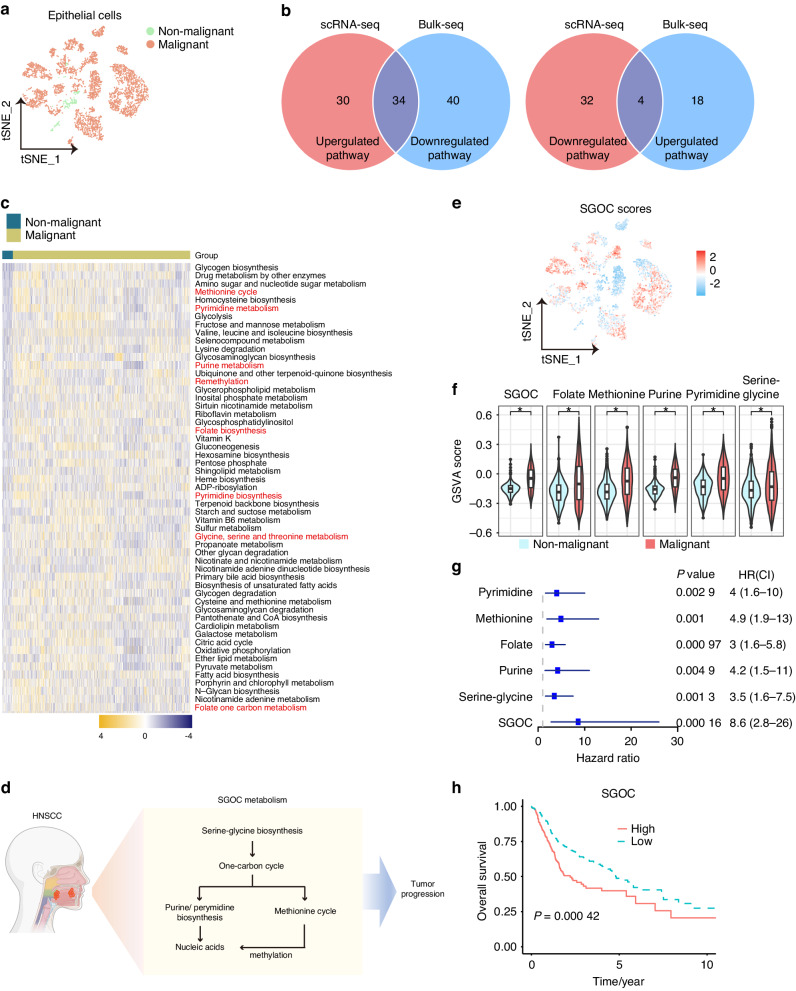
SGOC metabolism is upregulated and correlated with poor prognosis in HNSCC. **a** UMAP plots of epithelial cells in HNSCC samples colored by cell type (GSE181919). **b** Venn diagram of differentially expressed metabolic pathways identified by scRNA-seq and bulk-seq profiles (TCGA-HNSC). **c** Heatmap of differentially expressed metabolic pathways identified by scRNA-seq. **d** Sketch map of SGOC metabolic network. **e** UMAP plots of epithelial cells colored by SGOC cores. **f** Violin diagram showed the scores of SGOC metabolic pathways between non-malignant and malignant cells of scRNA-seq. **g** Univariate Cox analysis of the correlations between SGOC metabolic network and overall survival of HNSCC patients in TCGA-HNSC cohort. **h** Kaplan–Meier curves of overall survival (OS) according to SGOC scores in TCGA-HNSC cohort. *P < 0.05

Figure 1 illustrates the custom analysis pipeline utilized in the current study. The scRNA-seq profiles of 20 HNSCC patients (GSE181919) were analyzed, resulting in the clustering of a total of 47,711 cells into distinct cell types, including epithelial cells, fibroblasts, endothelial cells, myocytes, immune NK/T cells, B/plasma cells, macrophages, dendritic cells, and mast cells, based on the molecular markers of each cell type (Fig. S1a, b). Subsequently, the inferCNV method was employed to separate malignant cells from non-malignant cells with normal karyotypes (Fig. S1c).To elucidate the metabolic rewiring of HNSCC, non-malignant and malignant epithelial cells were selected, and the expression scores of 114 metabolic pathways, based on KEGG database,^13^ were quantified using GSVA algorithm (Fig. 2a). In comparison to non-malignant epithelial cells, 100 metabolic pathways across ten major metabolic classes exhibited differential expression (|GSVA *t*-value | > 2, adj.P < 0.05) in malignant cells, with 64 pathways upregulated and 36 downregulated (Fig. 2b, Fig. S2a, Table S1). Additionally, the expressions of all metabolic pathways in bulk tissues were calculated based on TCGA-HNSC dataset (Table S1). Remarkably, several metabolic alterations identified in malignant cells via scRNA-seq profiles were not observed in bulk tumor tissues. Furthermore, 38 of the 114 (33%) critical metabolic pathways for cell proliferation were upregulated in malignant cells as identified by scRNA-seq profiles, were significantly downregulated in bulk RNA-seq, including citric acid cycle, glycolysis, oxidative phosphorylation and fatty acid biosynthesis (Fig. 2b, Fig. S2b, c). This suggests that the metabolic reprogramming events defined by previous bulk tissue studies were largely obscured by the complex cellular components.

We initially observed substantial upregulation of the serine-glycine, folate, purine, pyrimidine, and methionine metabolic pathways within the SGOC metabolic network in malignant cells (Fig. 2c, d). To further delineate the rewiring of SGOC metabolism, we compiled gene lists associated with the SGOC metabolic network from GO and KEGG sources and quantified their expression scores (Table S2). Similarly, SGOC and its branched metabolic pathways were all more highly expressed in malignant cells compared to non-malignant keratinocytes, while the expressions of SGOC network showed no significant alteration in some bulk tumor tissue cohorts (Fig. 2e, f, Fig. S3a, b). Furthermore, univariate COX regression analysis and Kaplan–Meier survival analysis revealed that the SGOC network and its component pathways were all prognostic candidate pathways (Fig. 2g). HNSCC patients with higher SGOC scores exhibited shorter overall survival time (OS) than those with lower scores (Fig. 2h, Fig. S4). Thus, upregulation of SGOC may represent a prominent metabolic disorder in HNSCC and is associated with poor clinical outcomes.

### High SGOC metabolism contributes to aggressive phenotype in HNSCC

**Fig. 3:**
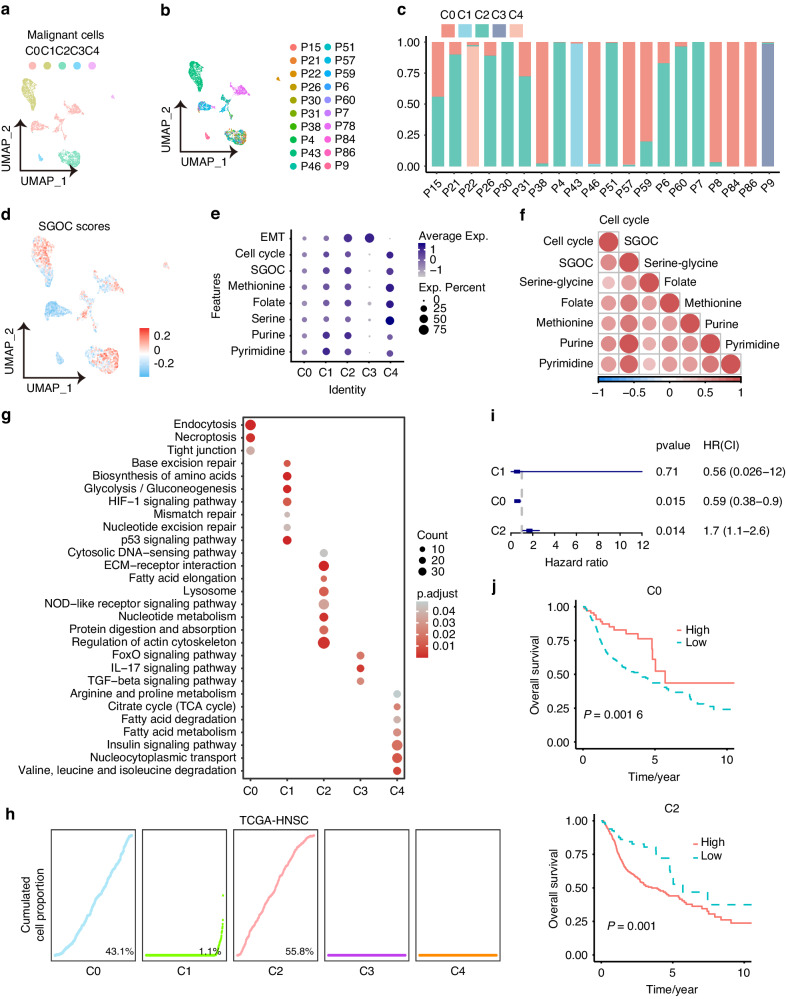
High SGOC metabolism shows an aggressive phenotype. UMAP plots of malignant cells colored by cell type (**a**) and patient origin (**b**). **c** The hierarchical clustering of the malignant clusters colored by patient origin. **d** UMAP plots of malignant cells colored by SGOC cores. **e** Dot-plots shows the expressions of SGOC metabolic network, EMT and cell cycle. **f** Spearman correlation analysis of the expressions of SGOC metabolic network and cell cycle. **g** KEGG analysis of the pathway enrichment of feature genes of malignant cell clusters. **h** The cumulative cell proportion of the malignant cell clusters is shown in TCGA-HNSCC. **i** Univariate Cox analysis of the correlations between malignant cell clusters and overall survival of HNSCC patients in TCGA-HNSC cohort. **j** Kaplan–Meier curves of overall survival (OS) according to malignant cell clusters in TCGA-HNSC cohort. *P < 0.05

To assess the transcriptional metabolic heterogeneity of HNSCC cancer cells, the malignant cells were re-classified into five sub-clusters based on their expression states (C0 - C4, Fig. 3a). The cluster composition varied greatly among different patients, demonstrating the transcriptional diversity of HNSCC (Fig. 3b, c). Then, we calculated the SGOC score of each cluster using GSVA. We found that each cluster exhibited a differential SGOC score, highlighting the high metabolic heterogeneity within malignant cells (Fig. 3d, e). Functional analysis based on GSEA hallmark demonstrated that clusters with higher SGOC scores (C1, C2 and C4) were associated with a higher cell cycle signature (Fig. 3e). The scores of SGOC pathways are positively correlated with cell cycle signature (Fig. 3f). Additionally, KEGG analysis indicated that clusters with high SGOC scores were enriched in cancer hallmarks, such as glycolysis and HIF-1 signaling (C1), nucleotide metabolism (C2), and nucleocytoplasmic transport (C4) (Fig. 3g).

Next, we conducted deconvolution analyses to evaluate the proportions of cell type in bulk RNA-seq data from TCGA-HNSCC, GSE41613, GSE65858, and GSE42743, identifying three sub-clusters in bulk tissues, which underscored the robustness of our classification (Fig. 3h, Fig. S5a).To determine which sub-cluster was associated with unfavorable clinical outcomes, univariate COX and Kaplan–Meier survival analysis were performed. The results supported that patients with high C0 (low SGOC scores) exhibited longer OS, while those with high C2 (high SGOC scores) showed shorter OS (Fig. 3i, j, Fig. S5b). Collectively, these findings demonstrate that SGOC metabolism serves as an indicator of high cellular aggressiveness in HNSCC.

### A novel 4-gene SGOC signature exhibits favorable prognostic prediction efficiency in HNSCC

**Fig. 4:**
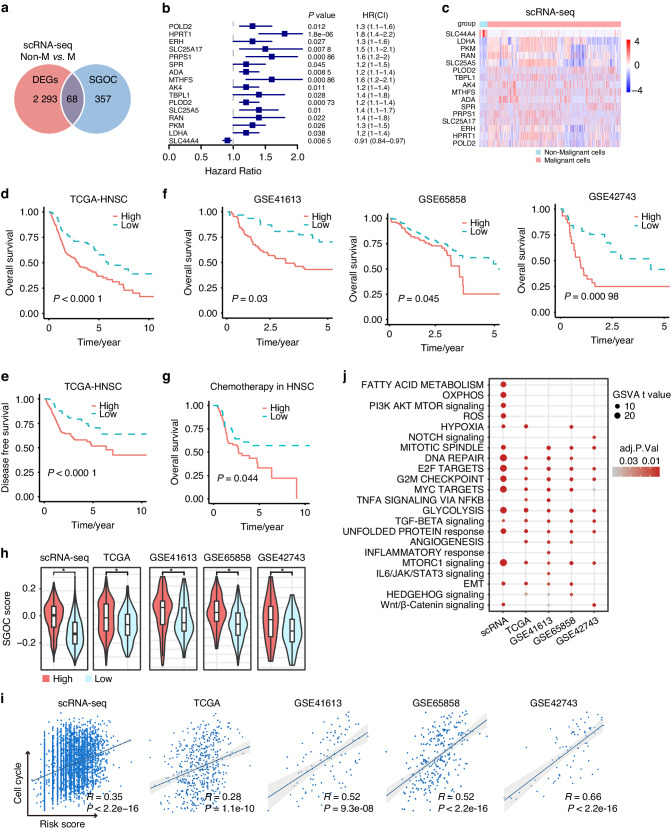
Developing a four-gene SGOC signature for prognostication in HNSCC patients. **a** Venn diagram of differentially expressed SGOC-associated genes between malignant cells and non-malignant cells. **b** Univariate Cox analysis of the correlations between SGOC-associated genes and overall survival of HNSCC patients in TCGA-HNSC cohort. **c** Heatmap of differentially expressed SGOC-associated genes between malignant cells and non-malignant cells. **d**, **e** Kaplan–Meier curves for the OS (**d**) and disease-free survival (DFS) (**e**) of high- and low-risk groups in the TCGA-HNSC cohort. **f** Kaplan–Meier curves for the OS of high- and low-risk groups in the GSE41613, GSE65858 and GSE42743 cohorts. **g** Kaplan–Meier curves for the OS of high and low-risk groups in patients treated with chemotherapy in TCGA-HNSC cohort. **h** Violin diagram showed the differences of SGOC scores between high- and low-risk patients. **i** Spearman correlation analysis of the SGOC risk scores and cell cycle scores. **j** Dot-plot shows the expressions of hallmark signaling of high- and low-risk patients. *P < 0.05

To enhance the clinical applicability of the SGOC metabolic network, we aimed to construct an SGOC-based signature for prognostic and treatment response prediction. Sixty-eight differentially expressed SGOC genes were identified through the intersection of differentially expressed genes (DEGs) between non-malignant and malignant cells (Fig. 4a). Among these, 16 candidate prognostic biomarkers associated with OS were figured out through univariate COX analysis (Fig. 4b, c, Table S3). Subsequently, we randomly divided 499 HNSCC patients from TCGA into training (*n* = 250) and testing (*n* = 249) cohorts. LASSO penalized cox regression analysis based on these candidates was performed, and a 4-gene SGOC prognostic signature was established in the training cohort (Fig. S6a−c). The Kaplan–Meier survival curve was used to assess the predictive power of the prognostic signature. Patients were stratified into high- and low-risk groups based on the best cut-off value determined using the ROC method. In the training, testing and total TCGA-HNSC cohorts, patients in the high-risk group exhibited shorter OS and disease-free survival (DFS) compared to those in the low-risk group (Fig. 4d, e, Fig. S6d−g). External cohorts (GSE65858, *n* = 270; GSE41613, *n* = 97; GSE42743, *n* = 74) validated that patients in the high-risk group exhibited poorer clinical outcomes (Fig. 4f). Furthermore, we explored the relationships between the SGOC risk score and OS in HNSCC patients who received chemotherapy, revealing that patients in the high-risk group had longer OS after chemotherapy treatment (Fig. 4g).

To determine whether the SGOC risk-score served as an independent prognostic factor for OS, potential predictors including gender, age, lymphovascular invasion, perineural invasion, HPV status, alcohol consumption, TNM stage, and risk score were analyzed via univariate cox regression in the TCGA-HNSC cohort (Table S4). Individual risk factors with a cox *p* < 0.05 (lymphovascular invasion, perineural invasion, HPV status, and SGOC risk-score) were further analyzed using multivariate cox regression. The results indicated that the SGOC risk-score, lymphovascular invasion, and perineural invasion were independent risk factors for OS in HNSCC (Table S4).

Meanwhile, we verified that patients with a high risk had higher SGOC scores (Fig. 4h). The risk scores were positively correlated to cell cycle (Fig. 4i). Additionally, oncogenic signaling and cancer hallmarks were further aggravated in high-risk patients, such as epithelial-mesenchymal transition (EMT), glycolysis, angiogenesis, DNA repair, TGFβ signaling, mTORC1 signaling, MYC and E2F targets (Fig. 4j). Taken together, we have demonstrated that the novel 4-gene SGOC signature might serve as a potential metabolic biomarker for prognosis prediction. Exploring the heterogeneity of SGOC metabolism may contribute to finding a precise therapeutic strategy for each HNSCC patient.

### High SGOC relates to low immune cell infiltration and poor response to immunotherapy

**Fig. 5:**
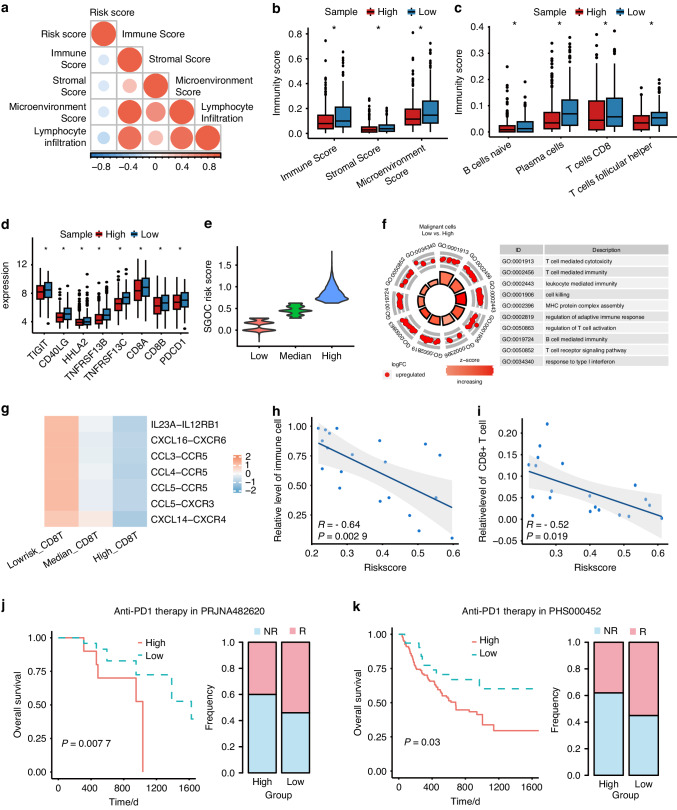
The distinct immunological characteristics based on SGOC metabolism. **a** Spearman correlation analysis of the SGOC risk scores and TME scores in TCGA-HNSC cohort. Boxplot shows the scores of TME (**b**), the expressions of immune cells (**c**) and ICIs (**d**) in the high- and low-risk groups of TCGA-HNSC cohort. **e** Malignant cells were divided into low, median and high according to SGOC scores. **f** GO enrichment analysis was performed on the differentially expressed genes between low-SGOC and high-SGOC malignant cells in scRNA-seq cohort. **g** Heatmap showing the interaction intensity between chemokines (CCL3/-4/-5, CXCL14/-16) and cytokines (IL23A) from malignant cells (SGOC-high, -low and -median) and the chemokine receptor (CXCR3/-4/-6, CCR5) and cytokines receptor (IL12RB1) on CD8 + T cells according to the CellCall analysis. Correlations between immune cells (**h**) or CD8 + T cells (**i**) infiltration and risk scores were determined by Spearman correlation analysis in scRNA-seq cohort. **j**, **k** Kaplan–Meier curves for the OS of high- and low- risk groups in anti-PD1 immunotherapy cohorts. *T* test, *P < 0.5

Next, we aimed to characterize the heterogeneity of the TME between high- and low-risk groups to explore potential immunotherapeutic vulnerabilities. In bulk tissues, numerous immune pathways were identified to be enriched in the low-risk group, including T cell co-stimulation and cytokine-cytokine receptor interaction (Fig. S7a, b). Subsequently, we explored the correlations between SGOC risk score and immune signals. We found that SGOC risk score was negatively correlated with immune score, stromal score, microenvironment score and lymphocyte infiltration (Fig. 5a). Low-risk patients showed higher immune score, stromal score and microenvironment score (Fig. 5b). The CIBERSORT and MCPcounter deconvolution algorithms indicated greater infiltration of immune cells in the TME of the low-risk group compared to the high-risk group, including CD8 + T cells and follicular helper T cells (Fig. 5c, Fig. S8a, b). Yet, the immune checkpoint inhibitors PD-1, HHLA2 and TIGIT were highly expressed in low-risk tumors, implying that the TME of low-risk patients was characterized by the accumulation of exhausted T cell infiltration (Fig. 5d).

To further validate the relationship between SGOC metabolism in malignant cells and the immune microenvironment, the scRNA-seq profiles were re-clustered into malignant, immune and stromal cells (Fig. S8c). The SGOC risk scores of malignant cells were calculated, based on which cells were segregated into SGOC-low, -medium or -high groups (Fig. 5e). The differentially expressed genes between SGOC-low and SGOC-high malignant cells were apparently enriched in immune-related biological functions, such as T cell activation, T cell mediated immunity, and T cell mediated cytotoxicity (Fig. 5f). To further validate the interactions between malignant cells and T cells, the T cells scRNA-seq profiles were re-clustered into CD8 + T cells, CD4 + T cells, naïve T cells, cycling T cells and NK T cells (Figure S8d). Analysis of the interactions between CD8 + T cells and SGOC-low, -medium, or -high group using the CellCall algorithm revealed that, in comparison with the SGOC-medium or -high group, SGOC-low cells had stronger chemotaxis toward T cells, indicating that SGOC-low cells in the TME mainly attracted T cells (Fig. 5g). Supporting this, the SGOC risk scores of malignant cells were negatively related to immune cells and T cell infiltration (Fig. 5h, i). In short, there is a strong possibility that SGOC metabolic heterogeneity might reflect different therapeutic responses to immunotherapy.

Therefore, we recruited the PRJNA482620 and PHS000452 immunotherapy cohorts to explore the relationship between SGOC risk score and therapeutic responses. Kaplan–Meier survival analysis confirmed that patients in the low-risk group had better OS than those in the high-risk group after anti-PD1/PD-L1 immunotherapy treatment. A higher proportion of patients responded to anti-PD1 treatment was identified in the low-risk group (Fig. 5j, k). Taken together, these results imply that compared with high-risk patients, low-risk patients had a more immunologically active TME and were more sensitive to immunotherapy. However, high-risk patients had a poor response to either chemotherapy or immunotherapy, indicating an urgent need to find a therapeutic target for high-risk patients.

### IMPDH1 is a SGOC metabolic target for provoking the malignant features of HNSCC cells

**Fig. 6:**
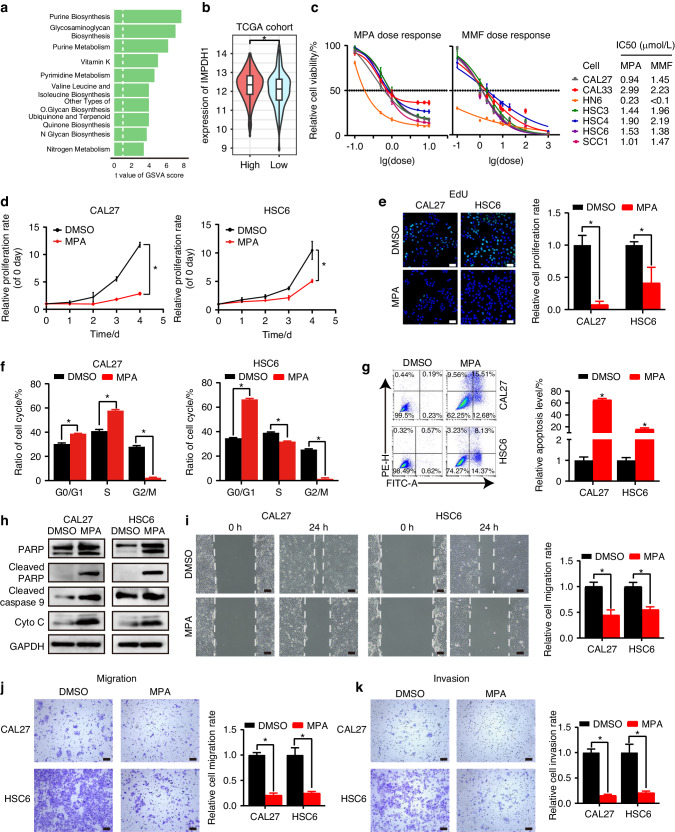
IMPDH inhibitor represses HNSCC cell progression in vitro. **a** The decentralized bar chart shows the differentially expressed metabolic pathways based on ssGSEA analysis between high- and low-risk groups in TCGA-HNSC dataset. **b** Violin plot of IMPDH1 expressions in high- and low-risk groups. **c** CCK8 was applied to test the sensitivities of HNSCC cells to different doses of MPA or MMF. The IC50s were calculated. After treating with DMSO or MPA (10 μmol/L), the cell viability of HNSCC cells were determined using CCK-8 (**d**), the proliferation rates were examined by EdU assay (**e**), the cell cycle (**f**) and apoptosis (**g**) were tested using flow cytometry, the protein levels of endogenous apoptosis markers were measured by western blot (**h**), the cell migration ablility was determined by Would healing assay (**i**) and Transwell assay without matrigel (**j**), and the invasion ability was detected by Transwell assay with matrigel (**k**). Mean ± s.d.; *P < 0.05; (**d**) two-way ANOVA, (**e**−**g**, **i**−**k**) Student’s *t* tests. Scale bars, (**e**) 50 μm, (**i**) 100 μm, (**j**, **k**) 200 μm

For the sake of identifying a potential therapeutic target, we elucidated the underlying metabolic mechanism between high- and low-risk groups. Twenty-two metabolic pathways were found to be upregulated in high-risk patients, among which purine synthesis was the most noticeably upregulated one (Fig. 6a, Table S5). IMPDH1, the rate-limiting enzyme of de novo purine synthesis, was substantially upregulated in the high-risk group (Fig. 6b). Then, we pharmacologically inhibited the IMPDH1 activity of HNSCC cells using a pan IMPDH inhibitor (MPA) and its pro-drug (MMF) to assess their anti-tumor effects.^14,15^ CCK8 assay showed that treatment with MPA or MMF dramatically decreased HNSCC cell viability (Fig. 6c, d). EdU assay demonstrated a direct inhibition of cancer cell proliferation by MPA (Fig. 6e). MPA treatment increased the proportion of cells in G0/G1 phase and concomitantly decreased the proportion of cells in G2/M phase (Fig. 6f). Moreover, the proportion of apoptotic cells and the expressions of apoptotic markers (cleaved-PARP, cleaved-caspase-9, cytochrome-c) were increased by MPA (Fig. 6g, h). The wound healing and Transwell assays revealed that MPA notably suppressed the migration and invasion capabilities of HNSCC cells (Fig. 6i, k). Collectively, these results demonstrate that IMPDH-mediated purine biosynthesis is a promising metabolic target for high-risk HNSCC patients, pharmacological inhibiting of this pathway might provide a novel therapeutic strategy.

### Inhibiting IMPDH1 represses cancer cell progression via triggering GTP-exhaustion nucleolar stress in HNSCC

**Fig. 7:**
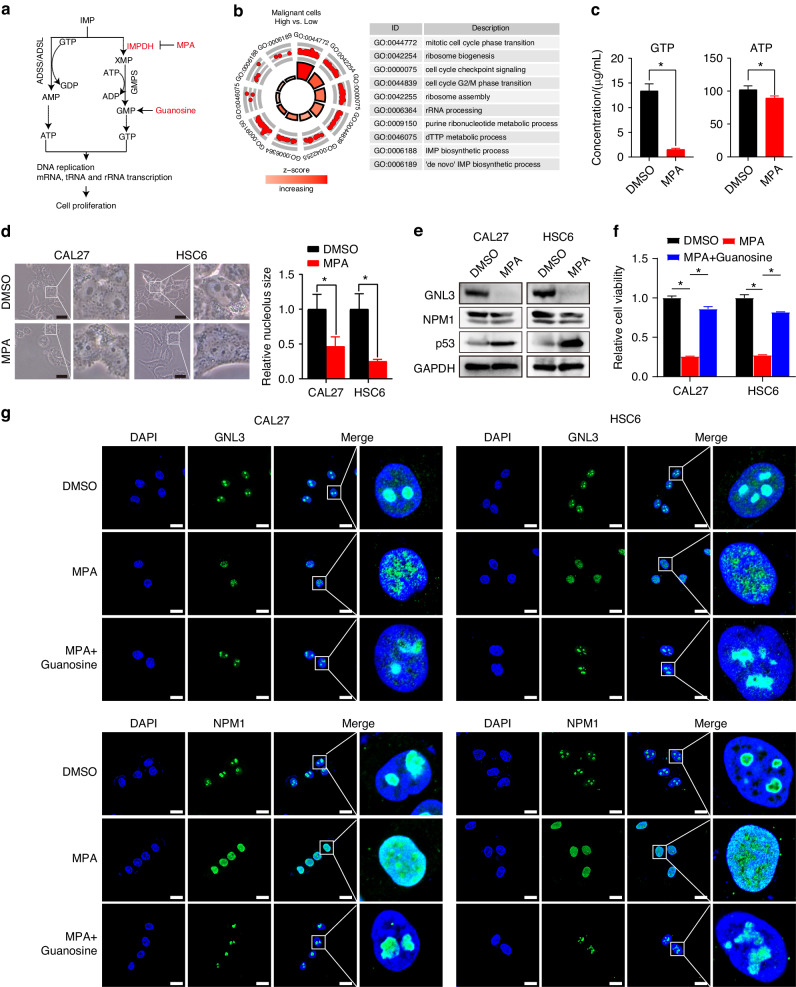
IMPDH inhibitor represses HNSCC cell progression through provoking GTP-exhaustion nucleolar stress. **a** Sketch map of de novo purine synthesis pathway. **b** GO enrichment analysis was performed on the differentially expressed genes between low-SGOC and high-SGOC malignant cells in scRNA-seq cohort. **c** LC was performed to measure the GTP and ATP contents in CAL27 cells treated with DMSO or MPA. **d** Effects of MPA treatment on nuclear size and morphology of HNSCC cells. **e** The protein levels of nucleolar stress markers were measured by western blot. GAPDH was used as the loading control. After treating with DMSO, MPA (10 μmol/L), or MPA and guanosine (100 μmol/L), and cell viability was analysed by CCK-8 assay (**f**), the localizations of GNL3 and NPM1 were determined using immunofluorescence imaging (**g**). Mean ± s.d.; *P < 0.05; (**c**, **d**) Student’s *t* tests, (**f**) one-way ANOVA. Scale bars, (**d**) 40 μm, (**g**) 20 μm

To further investigate the tumor-suppressive effects of the IMPDH inhibitor in HNSCC, we explored its underlying mechanisms. IMPDH1 has been known to support cell proliferation by generating purine nucleotides for DNA replication, RNA (mRNA, tRNA and rRNA) transcription^16,17^ (Fig. 7a). Similarly, the differentially expressed genes between SGOC-high and SGOC-low malignant cells were enriched in purine nucleotide synthesis, ribosome synthesis and cell cycle (Fig. 7b). Then, we detected the effects of MPA on ATP and GTP contents using liquid chromatography, which confirmed that MPA prominently inhibited GTP production and weakly inhibited ATP production (Fig. 7c). After treatment with MPA, the nucleoli of HNSCC cells severely shrank, indicating induced nucleolar stress (Fig. 7d). Nucleolar stress is characterized by morphological and functional alterations of the nucleolus and cause molecular changes, including degradation and delocalization of NPM1 and GNL3 and stabilization of p53, which in turn contributes to activation of pathways that promote cell cycle arrest or apoptosis.^14,18,19^ Western blotting confirmed that MPA treatment degraded the nucleolar proteins (NPM1 and GNL3), and increased p53, supporting a nucleolar stress induced by MPA-mediated inhibition of de novo purine biosynthesis (Fig. 7e).

**Fig. 8:**
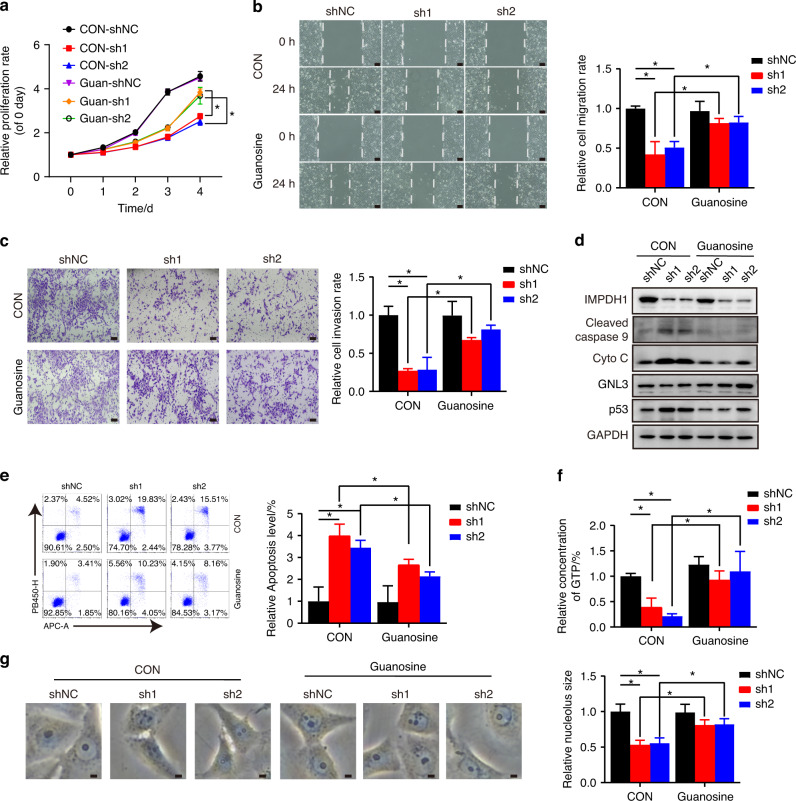
Knockdown IMPDH1 represses HNSCC cell progression in vitro. **a** After treating with DMSO or guanosine (100 μmol/L), the cell viability of HSC6 IMPDH1shs cells were determined using CCK-8. The cell migration ablility was determined by Would healing assay (**b**) and the invasion ability was detected by Transwell assay with matrigel (**c**). The protein levels of endogenous apoptosis markers and nucleolar stress markers were measured by western blot (**d**). The apoptosis was tested using flow cytometry (**e**). **f** LC was performed to measure the relative GTP contents in HSC6 IMPDH1shs cells treated with DMSO or guanosine. **g** Effects of IMPDH1 knockdown on nuclear size and morphology of HNSCC cells. Mean ± s.d.; *P < 0.05; two-way ANOVA. Scale bars, (**b**, **c**) 100 μm, (**g**) 5 μm

Hence, we added exogenous guanosine (100 μmol/L) to MPA-treated HNSCC cells to counteract the effect of the IMPDH inhibitor. CCK-8 assay results showed that cell viability, originally suppressed by MPA, was rescued by exogenous guanosine (Fig. 7f). Furthermore, the delocalization of nucleolar protein (NPM1 and GNL3) induced by MPA was reversed by guanosine supplementation, as evidenced by the relocation of NPM1 and GNL3 to nucleolus (Fig. 7g). These data provide evidence that IMPDH inhibitor suppresses HNSCC cell progression by prompting GTP-exhaustion nucleolar stress.To further elucidate the biological roles of IMPDH1 in HNSCC, we established HNSCC cells with stable knockdown of IMPDH1 (Fig. S9). Cellular biological function experiments demonstrated that, akin to the effects of MPA, the depletion of IMPDH1 inhibited HNSCC cell viability, migration, invasion, and induced GTP-exhaustion nucleolar stress and apoptosis. Importantly, these effects of IMPDH1-shs were reversed by the addition of exogenous guanosine (Fig. 8). Therefore, these findings underscore the potential of inhibiting IMPDH1-mediated purine biosynthesis to impede HNSCC progression.

### IMPDH inhibitor suppresses HNSCC tumor growth in vivo

**Fig. 9:**
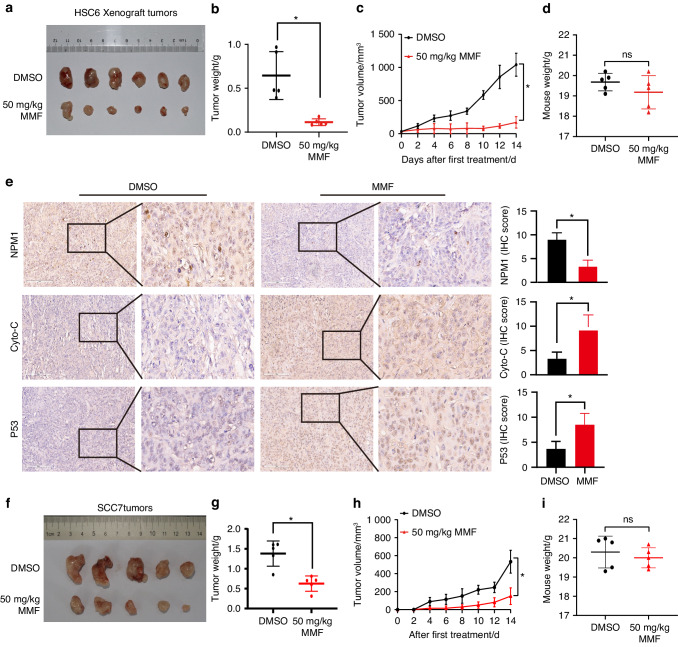
IMPDH inhibitor represses HNSCC tumor growth in vivo. **a** Representative images of primary tumors appearance in nude mice. The weights (**b**) and volumes (**c**) of primary tumors taken from node mice. **d** The weights of nude mice. **e** Pictures and quantifications of NPM1, cytochrome-c, and P53 expressions examined by IHC staining in tumor sections of nude mice. **f** Representative images of primary tumors appearance in nude mice. The weights (**g**) and volumes (**h**) of primary tumors taken from node mice. **i** The weights of nude mice. Mean ± s.d.; ns: not significant, *P < 0.05, compared with DMSO treatment; Student’s *t* tests

The in vivo pharmacological roles of the IMPDH inhibitor on HNSCC growth were determined using a subcutaneous tumor model in nude mice. Following two weeks of treatment with the MPA prodrug (MMF, 50 mg/kg), mice were euthanized, and the subcutaneous tumors were harvested. Compared to the tumors in the DMSO group, those in the MMF treatment group exhibited slower growth rates, smaller tumor volumes, and lower tumor weights (Fig. 9a−c). Remarkably, there was no significant difference in body weight between two groups, indicating a low incidence of side effects associated with MMF treatment (Fig. 9d). Additionally, the levels of nucleolar stress and apoptosis biomarkers (NPM1, P53 and cytochrome-c) were assessed. In contrast to the DMSO treatment group, the MMF treatment group showed reduced NPM1 levels and higher levels of P53 and cytochrome-c (Fig. 9e).

Furthermore, a subcutaneous model was established using immune-competent C3H mice to evaluate the impact of MMF on anti-tumor immunity. After two weeks of MMF treatment (50 mg/kg), the subcutaneous tumors and spleens of C3H mice were dissociated, and the immune cells within the tissues were isolated. Results demonstrated that MMF significantly attenuated tumor growth, leading to reduced tumor volumes and weights, with no significant effects observed on body weight (Fig. 9f−i). Flow cytometry analysis revealed no significant differences in the proportions of tumor-infiltrating CD8 + T cells or the expression levels of IFN-γ, PD-1, and LAG-3 between the MMF and DMSO groups, indicating minimal effects of MMF on CD8 + T cell infiltration and activation (Fig. S10a–d). Overall, these findings highlight the IMPDH inhibitor’s capacity to effectively suppress HNSCC tumor growth in vivo while demonstrating a low incidence of side effects.

## Discussion

To sustain aggressive behaviors and adapt to the complex and changing microenvironment, cancer cells undergo plentiful metabolic adaptations.^20^ Targeting specific metabolic phenotypes presents vulnerabilities to anti-cancer treatment and yields varied clinical outcomes.^21,22^ Abundant metabolic molecules have been identified and have shown promising efficacy in halting tumor progression in preclinical studies.^23,24^ However, the unsatisfactory efficacy of metabolic therapies in clinical trials underscores the urgent need to recognize the flexibility and intricacy of the metabolic network in human cancers. HNSCC is one of the most aggressive human cancers and harbours numerous metabolic alterations, as reported in our previous work and others.^12,20^ Yet, the currently identified metabolic alterations in HNSCC tumours have mostly been based on bulk tissue studies. The complex cellular compositions of bulk tissues seriously hinder the accurate identification of metabolic alterations in malignant cells. Recent advances in scRNA-seq greatly facilitate the study of individual cell populations and enable a multidimensional exploration of intra- and inter-tumour metabolic heterogeneity. Here, we leveraged the scRNA-seq profiles to explore the metabolic heterogeneity of malignant cells in HNSCC. Several metabolic alterations in malignant cells that were previously overlooked were identified in scRNA-seq profiles, including the SGOC network. Consequently, the significant differences in results obtained by these two methods suggest a complementary relationship, enabling the redefinition of intra- and inter-tumour metabolic heterogeneity and its influence on the TME of HNSCC. Although we observed apparent upregulation of the branched metabolic pathways of the SGOC network in malignant cells, the glycine-serine-threonine metabolism was found to be downregulated in bulk tissues, significantly hindering the study of its roles in HNSCC progression. Sub-clusters of malignant cells from a single patient exhibited distinct expression patterns of the SGOC network, indicating its flexible role in adapting to the complex TME and regulating tumor progression. Despite the intra-tumor heterogeneity, HNSCC patients with high SGOC expressions experienced unfavourable clinical outcomes. Recently, the oncogenic roles of SGOC metabolism have been highlighted due to its crucial roles in maintaining the aggressive behaviors of malignant cells, such as uncontrolled proliferation, metastasis, chemotherapy resistance, and immune evasion.^25–27^ For example, Increased SGOC genes expressions in colorectal cancer could facilitate tumor progress.^28^ Transcriptional profiling revealed that high-risk human neuroblastomas acquired a metabolic program characterized by transcriptional activation of the serine-glycine synthesis pathways, leading to poor clinical outcome.^29^ Moreover, enhanced serine biosynthesis pathway is associated with drug resistance of melanoma, pancreatic, and non-small cell lung cancers.^30^ In oral squamous cell carcinoma (OSCC), the imbalance in the amino acid and purine metabolic pathway was reported to affect patients’ prognosis.^31^ In the present study, we characterized that the SGOC network was associated with the aggressive features of HNSCC malignant cells, such as cell cycle and several oncogenic signaling pathways, demonstrating its essential roles in tumorigenesis.

Growing evidence has indicated that genes of the SGOC metabolism can serve as prognostic markers and potential therapeutic targets for tumor treatment.^32^ To better understand the clinical application potential of the SGOC network in HNSCC, we developed a novel classifier of a 4-gene SGOC prognostic signature (PRT1, TBPL1, PLOD2, and SLC44A4). HPRT1, which plays a central role in the generation of purine nucleotides through the purine salvage pathway, has been identified as an unfavourable prognostic marker in many cancers, including HNSCC.^33^ TBPL1 encodes a member of the TATA box-binding protein family, activating the transcription of metabolic genes. PLOD2 catalyses the hydroxylation of lysyl residues and could promote the metastasis capacity of gastric cancer, sarcoma and colorectal cancer^34–36^ However, SLC44A4, which was reported to be upregulated in prostate and pancreatic cancers,^37^ was downregulated in malignant cells and correlated with a good prognosis for HNSCC, indicating an undefined role in tumor suppression that needs further elucidation. Our subtyping system based on the 4-gene SGOC prognostic signature could separate patients with aggressive tumours and provide valuable information about clinical values in prognosis and treatment responses. Patients with lower SGOC risk scores had better clinical outcomes.

Immune checkpoint inhibitors (ICIs), represented by PD-1/PD-L1, have ushered in a new era of immunotherapy for HNSCC. Nevertheless, a significant proportion of patients fail to respond to ICIs. Therefore, it is necessary to find biomarkers to assist in decision-making regarding immunotherapy selection. The dysregulated SGOC metabolism facilitates cancer cell immune evasion and affects tumour microenvironment via its products and intermediates. For example, 2'3’-cyclic GMP-AMP (cGAMP) is a critical metabolite in activating the innate immune STING pathway. Depletion of extracellular cGAMP reduces immune cell infiltration and lead to immune escape.^38^ High levels of ATP could activate dendritic cells in the TME, promoting antigen presentation and antitumor immunity.^39^ Adenosine, generated by the degradation of extracellular ATP, acted as an immune suppressor by inhibiting the proliferation of effector T lymphocytes and the secretion of inflammatory cytokines.^40^ This study revealed that HNSCC patients with low SGOC metabolism had more CD8^+^ T cells infiltration, and showed better responses to either chemotherapy or immunotherapy, whereas patients with higher SGOC risk scores had worse responses to current treatment therapies, potentially due to the upregulation of IMPDH1-mediated purine synthesis.

Targeting metabolic vulnerabilities could increase the specificity and sensitivity of established therapies, and has garnered significant interest recently.^41^ Cancer cells upregulate the IMPDH-mediated purine synthesis to meet the increasing demand for DNA replication, rRNA transcription, and ribosomal biogenesis, blocking which could induce nucleolar stress.^41^ For example, enhancement of de novo purine biosynthesis was considered as a major driver of chemoresistance in glioblastoma, which could be repressed by the IMPDH inhibitors.^14,15,42^ MPA and MMF have been shown to inhibit cell proliferation, induce differentiation, or apoptosis in many cancers, such as non-small cell lung adenocarcinoma, colon cancer, and leukemia. However, their anti-tumor effects in HNSCC are still unknown. Our findings emphasize that inhibition of IMPDH could repress the proliferation, migration and invasion capabilities of HNSCC cells by decreasing GTP production, expanding the scope of IMPDH1 inhibitors in anti-tumor therapy.

**Fig. 10:**
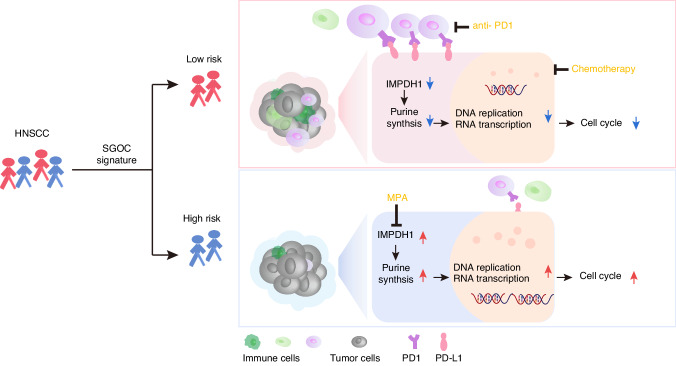
Graphical summary of stratifying HNSCC based on SGOC metabolism heterogeneity

Our study illuminates the pivotal role of the SGOC metabolism in the progression and immune microenvironment modulation of HNSCC. We introduced a novel molecular classification system for HNSCC based on the intrinsic heterogeneity of SGOC metabolism, revealing its significance as a prognostic indicator and therapeutic target. High SGOC metabolism was associated with aggressive tumor behavior and resistance to current treatment modalities, while low SGOC metabolism correlated with better treatment response, particularly to chemotherapy and immunotherapy. Additionally, our findings elucidate the dysregulation of IMPDH1-regulated purine biosynthesis in high-risk patients, presenting an opportunity for targeted therapy using IMPDH inhibitors to inhibit tumor progression (Fig. 10).

In conclusion, this comprehensive understanding of SGOC metabolism provides valuable insights into tumor and tumor microenvironment heterogeneity in HNSCC, paving the way for refined molecular stratifications and the development of tailored therapeutic approaches. By targeting metabolic vulnerabilities, we can potentially enhance the efficacy of existing therapies and offer novel avenues for the treatment of HNSCC.

## Materials and methods

### Data collection

The scRNA-seq profiles, including normal tissues (*n* = 9), tumor tissues (*n* = 20), and lymph nodes (*n* = 4), were downloaded from GSE181919. Gene expression data and related clinical information for TCGA-HNSC (normal = 44, tumor = 499) were obtained from UCSC Xena, with one patient (TCGA-CQ-A4CA-01) excluded due to missing survival data. For verification, datasets GSE41613, GSE65858, GSE42743, GSE30784, GSE25099, GSE37991 and GSE31056 were employed. Additionally, GBM-PRJNA482620 and Melanoma-PHS000452 from the TIGER database were acquired for immunotherapy studies. All expression data were log2 transformed.

### Quality controlling, processing and clustering of scRNA-seq data

Quality control and cell type annotation for GSE181919 were conducted following methods described in the corresponding article.^43^ In brief, the “Seurat 4.0” R package was used to filter the raw data of the gene expression matrix, resulting in the inclusion of a total of 47,711 cells for subsequent analysis. Batch effects of samples were corrected using “RunHarmony” in the “Harmony” R package. Dimension reduction and clustering were performed using UMAP with the “RunUMAP” and “FindClusters” functions. Cell types were annotated manually based on marker genes calculated by “FindAllMarkers” function. The “inferCNV” R package was exploited to distinguish malignant cells from epithelial cells of tumor tissues by calculate copy number variations (CNVs).

### Clarification of SGOC metabolic heterogeneity

For tumor metabolic heterogeneity analysis, single-sample gene set enrichment analysis (ssGSEA) was carried out using the “GSVA” package. The differential expressions of 114 metabolism pathways between non-malignant and malignant epithelial cells, as well as normal and tumor tissues, were calculated using the “Limma” package. Next, 425 genes involved in serine, glycine, one-carbon, folate, methionine, purine, and pyrimidine were downloaded from the Molecular Signature Database (MSigDB) and used to construct the SGOC metabolic network with five branched pathways^44–46^ (Table S2).

Malignant cells were classified into five groups using UMAP with the “RunUMAP” and “FindClusters” functions. The DEGs of the clusters were filtered using a threshold of adjusted *P*-values of < 0.05 and |log_2_FC | > 0.20. Kyoto encyclopaedia of genes and genomes (KEGG) pathway analysis was performed using the ‘clusterProfiler’ R package to evaluate the enrichment pathways of the sub-clusters. Then, deconvolution of the bulk RNA-Seq datasets were was performed using “MuSiC” R package.^47^ Univariate Cox regression analysis and Kaplan–Meier (K-M) survival curve analyses were conducted to evaluate prognostic value using the ‘survminer’ and ‘survival’ R packages.

### Construction of a prognostic SGOC-related gene signature

The differentially expressed SGOC metabolic genes between non-malignant and malignant cells in scRNA-seq data were calculated by the “Findmarkers” algorithm with the same threshold as mentioned above. The prognostic values of the dysregulated SGOC genes were determined using univariate Cox regression analysis. Subsequently, 13 dysregulated prognostic SGOC genes (*P* < 0.05) were selected to construct a prognostic signature through LASSO-Cox regression analysis using the ‘glmnet’ R package. Penalty parameter lambda (λ) of the model was determined by 10-fold cross-validation. The risk score of each patient was calculated according to the normalized expression of the candidate genes (Expi) and their corresponding regression coefficients (Coei). The formula for the risk score was constructed as follows:

$${\rm{Risk}}\;\, {\rm{score}}=\mathop{\sum }\limits_{i=1}^{N}({Expi}\times {Coei})$$

The final formula is as follows: Risk Score= 0.256 × PLOD2 + 0.251 × HPRT1 + 0.407 × TBPL1-0.112 × SLC44A4.

The risk score was calculated for each HNSCC patient, and based on this score, patients were stratified into high-risk and low-risk groups using an optimal cutoff value. Kaplan−Meier survival curve analysis was then conducted to evaluate the prognostic value of the SGOC gene signature using the ‘survminer’ and ‘survival’ R packages.

### Functional enrichment analysis of high- and low- risk HNSCC patients

DEGs between high- and low-risk groups in the TCGA-HNSC cohort were calculated using the R packages “limma”. For scRNA-seq data, malignant cells were extracted, and the risk score was calculated. The top 30% of risk scores were defined as the high risk group, while the bottom 30% were defined as the low risk group. DEGs between high- and low-risk groups in the scRNA-seq cohort were calculated using the R packages “FindMarkers”. The ssGSEA was carried out to analyze the enrichment of metabolism pathways. GO and KEGG pathway analysis were performed using the ‘clusterProfiler’ R package. The P-values of ssGSEA, GO terms and KEGG pathways were corrected. The correlation between SGOC-risk score and cell cycle score was calculated using Spearman correlation analysis.

The stromal score, immune score, ESTIMATE score and tumor purity score of each sample were computed using the “ESTIMATE” R package. The xCell score calculated by “xcell” R package provides a comprehensive tumor microenvironment landscape.^48^ The levels of immune cells in the TME were estimated by the CIBERSORT, xCell, and MCPcounter algorithms. Additionally, the correlations between the risk score and immune score, or immune cells infiltration, were calculated using Spearman correlation analysis, and the results were plotted by the “corrplot” R package.

### Cell-cell communication

T cells were re-clustered to perform the analysis of cell-cell communication through “RunHarmony”, “RunUMAP” and “FindClusters” functions. CD8 + T cells, high, low and median risk malignant cells were selected and “CellCall” algorithm^49^ was used to analyze the intercellular communication.

### Cell culture

Human HNSCC cell lines (HSC3, HSC4, HSC6, Cal33, CAL27, SCC1, HN6) were maintained in our laboratory in Guangzhou, China.^12,50,51^ CAL27, CAL33, HSC3, HSC6, and SCC1 cells were cultured in Dulbecco’s modified Eagle’s medium (DMEM; Gibco) supplemented with 10% fetal bovine serum (FBS; Gibco, USA). HN6 cells were grown in DMEM/F-12 (Gibco, USA) supplemented with 10% FBS. HSC4 cells were cultured in Minimum Essential Medium (MEM; Gibco, USA) supplemented with 10% FBS. The following inhibitors were used in this study: mycophenolic acid (MPA, Selleck, USA), mycophenolate mofetil (MMF, Selleck, USA).

### Plasmid construction and transfection

HNSCC cells with stable knockdown of IMPDH1 were generated as previously reported.^12,50^ The plko.1-shNC-GFP-puro, plko.1-IMPDH1-sh1-GFP-puro, and plko.1-IMPDH1-sh2-GFP-puro plasmids were obtained from Long Bioscience (Guangzhou, China). Briefly, the IMPDH1-shs or shNC plasmids were co-transfected with lentivirus packaging plasmids psPAX2 and pMD2.G into 293FT cells using polyethylenimine. Subsequently, lentiviral particles were collected and used to infect HSC6 cells. The stably transfected cells with shNC or IMPDH1-shs were selected using puromycin (10 mg/ml) (Sigma). The efficiency of knockdown was evaluated by quantitative real-time PCR and western blotting assays.

### Quantitative real-time PCR assay (qPCR)

The mRNA level of IMPDH1 was measured using qPCR. The SYBR Green-based qPCR analysis was conducted with the Light-Cycler 96 system (Roche). GAPDH served as an endogenous control for IMPDH1. The relative expression levels were calculated using the comparative threshold cycle equation (2 -ΔΔCT). The primer sequences were used as follows: IMPDH1, Forward Primer: 5’-CAGCAGGTGTGACGTTGAAAG-3’; Reverse Primer: 5’-AGCTCATCGCAATCATTGACG-3’. GAPDH, Forward Primer: 5’-CTCCTCCTGTTCGACAGTCAGC-3’, Reverse Primer: 5’-CCCAATACGACCAAATCCGTT-3’.

### CCK-8 assay

For cell viability assessment, the CCK-8 assay was applied. Approximately 1.5 × 10^3^ cells per well were seeded in 96-well plates. Following incubation with either DMSO or MPA treatment for the indicated times (0, 1, 2, 3 and 4 days), the cell medium was discarded, and cells were exposed to a mixture of 10 μL CCK8 (Sigma-Aldrich, USA) and 100 μL serum-free medium for an additional 2 h before detection. Cell viability was recorded based on absorbance readings at 450 nm using a spectrophotometric plate reader (Biotek, USA).

### EdU click chemistry assay and fluorescence imaging

Briefly, 1 × 10^5^ cells per well were seeded in confocal dishes. Following incubation with either DMSO or MPA treatment for 24 h, cells were exposed to 1 μL EdU (Beyotime, China). Then, cells were washed with phosphate-buffered saline (PBS, Sigma-Aldrich, USA), fixed in a 4% paraformaldehyde solution, permeabilized with 0.3% Triton-X 100 in PBS, and subjected to incubation with a reaction cocktail containing the necessary compounds for bonding of Alexa Fluor® 488 azide with EdU. Finally, cell nuclei were stained with DAPI (Sigma-Aldrich, USA). Cell imaging was performed using a laser scanning microscope (LSM 980, ZEISS).

### Flow cytometry analysis

For cell cycle detection, CAL27 and HSC6 cells treated with either DMSO or MPA were seeded into 60 mm dishes. Once cell confluence reached 80%, the cells were fixed in 70% cold ethanol and then subjected to testing with the Cell Cycle Detection Kit (KeyGEN, China). The fluorescence signals were recorded using flow cytometry (Cytoflex, Beckman Coulter, USA).

For the apoptosis assay, CAL27 and HSC6 cells treated with DMSO, MPA or guanosine were seeded in 6-well plates. After incubating with serum-free medium for 24 h, the cells were harvested. An Annexin VFITC/ PI Apoptosis Detection Kit (KeyGEN, China) was used to detect apoptotic cells. HSC6 IMPDH1-shs or shNC cells were dyed by Annexin V-APC/DAPI Apoptosis Kit (Procell, China). The flow cytometer was performed to count these apoptotic cells.

For detecting the expressions cytokines of CD8 + T cells, tumor or spleen tissues derived from C3H mice were grinded into single cells. Then, cells were stimulated in vitro with cell stimulation cocktail (1:500, TNB-4975-UL100, Tonbo Biosciences) for 5 h at 37 °C with 5% CO_2_, followed by PI staining for 15 min and surface markers staining for 30 min in the dark. Next, cells were fixed and permeabilized with intracellular fixation and permeabilization buffer, and stained with intracellular cytokine antibodies according to the manufacturer’s instructions. The following antibodies were used: CD8α (FITC conjugated, 53-6.7, Cat#100706, Biolegend), CD3e (APC conjugated, 145-2C11, Cat#100312, Biolegend), PD-1(Percp-Cy5.5 conjugated, 29 F.1A12, Cat#135208, Biolegend), LAG3 (Bv421-Conjugated, C9B7W, Cat#125221, Biolegend), Tim-3 (PE-Cy7 conjugated, RMT3-23, Cat#25-5870-82, eBioscience), IFN-γ (APC-eFlour 780 conjugated, XMG1.2, Cat#47-7311-82, eBioscience), TOX (PE-conjugated, TXRX10, Cat#12-6502-80, eBioscience).

### Western blotting

Cells were washed with ice-cold PBS and lysed with RIPA strong lysis buffer (Sigma-Aldrich, USA) supplemented with 1% protease and 1% phosphatase inhibitors (Beyotime, China). Then, 5× loading buffer (Beyotime, China) was added to the protein samples and cooked at 99°C for denaturation. The lysates were loaded onto 10% SDS-PAGE gel for separation and transferred to a 0.22 μm PVDF membrane (Millipore, USA). After blocking in 1× Protein Free Rapid Blocking Buffer (EpiZyme), the membranes were incubated with primary antibodies at 4 °C overnight, followed by incubation with species-matched secondary antibodies. Finally, the antigen-antibody reaction was tested by enhanced chemiluminescence (ThermoFisher, USA). The following antibodies were used: GAPDH (60004-1, 1:3 000, Proteintech), p53 (10442-1-AP, 1:1 000, Proteintech), B23/NPM1 (60096-1, 1:1 000, Proteintech), GNL3 (67169-1, 1:1 000, Proteintech), Cytochrome-C (66264-1, 1:2 000, Proteintech), rabbit IgG HRP-linked (7074, 1:3 000, CST), mouse IgG HRP-linked (7076, 1:3 000, CST), Cleaved PARP (5625, 1:1 000, CST), PARP (9532, 1:1 000, CST), Cleaved caspase-9 (20750, 1:1 000, CST), IMPDH1 (861791, 1:500, Zen BioScience).

### Migration and invasion assays

Cells were scratched using a 10 μL pipette after the cells in the six-well plate. Photos were taken at 0 h and 24 h after scratching. To evaluate the migration capability, 6 × 10^4^ CAL27 or HSC6 cells in 100 μL serum-free medium were added to the upper layer of transwell chamber (Corning, USA). The lower chamber of transwell was supplemented with DMEM medium with complete serum-medium. After 18 h for migration and 24 h for invasion, cells on the lower surface were fixed with 4% paraformaldehyde solution, stained with 0.4% crystalviolet (Beyotime, China), and counted under a microscope.

### Immunofluorescence assay

Cells were harvested and fixed with 4% paraformaldehyde. After permeabilization using 0.3% Triton-X 100 in PBS, cells were incubated with the primary antibodies at 4 °C overnight. Then, cells were stained with species-matched fluorescent secondary antibodies. Nuclei were stained with DAPI. The slides were viewed using a laser scanning microscope. The following antibodies were used: B23/NPM1 (60096-1, 1:200, Proteintech), GNL3 (67169-1, 1:200, Proteintech), Donkey anti-Mouse IgG (H + L) Alexa Fluor Plus 488 (A32766, 1:1 000, ThermoFisher).

### GTP and ATP content measurement

CAL27 or HSC6 cells treated with DMSO, MPA or guanosine were seeded in 60 mm dishes and subsequently harvested. Then cells were incubated with 80% cold carbinol (V/V) at 4 °C and collected. After centrifugation, the supernatant was collected and dried by a vacuum centrifugal concentrator at −50 °C to collect the precipitate. Then, the samples were dissolved in 60% acetonitrile before detecting the ATP and GTP content using a Triple quadrupole LC/MS.

### In vivo tumor models

All animal research procedures were carried out in strict accordance with the detailed rules of the Institutional Animal Care and Use Committee of Sun Yat-Sen University, with approval numbers 2023000092 and 2023003617. A total of 1 × 10^6^ HSC6 cells were subcutaneously injected into twelve female BALB/c nude mice (4–6 weeks old), while 5 × 10^6^ SCC7 cells were subcutaneously injected into twelve female C3H mice (4 weeks old). Upon reaching tumor volumes of approximately 40 mm^3^, the mice were randomly divided into 2 groups and intraperitoneally injected with either DMSO or MMF every 2 days. Accordingly, the tumor volumes were recorded. After 2 weeks of injection and tumor growth, the mice were sacrificed, and primary tumors and spleens were collected.

### Immunochemistry (IHC) analysis

IHC was performed on xenograft mice tissues.^50^ Briefly, dewaxing was carried out using xylene, followed by rehydration using alcohol with a gradient concentration. Endogenous peroxidase activity was blocked by 3% H_2_O_2_. Then, the slices were subjected to citrate-mediated high-temperature antigen retrieval. Afterward, slides were blocked with goat serum and incubated with primary antibodies overnight. Following washing with TBST solution, slides were incubated with secondary antibodies at room temperature. The Apreio AT2 digital whole slide scanner (Leica, Wetzlar, Germany) was applied to scan the slices. The following antibodies were used: B23/NPM1 (60096-1, 1:200, Proteintech), Cytochrome-C (66264-1, 1:2 000, Proteintech), p53 (10442-1-AP, 1:1 000, Proteintech).

### Statistical analysis

The bioinformatics and statistical analyses were conducted using R 4.3.1, SPSS 27 and GraphPad Prism 8.0 softwares. The Student’s *t* test or ANOVAs (one- or two-way) was used to preform statistical analyses. Data presented as the mean ± SD were extracted from at least three independent experiments. *P* < 0.05 was considered as significant.

### Supplementary information

Supplementary Figures

Supplementary Tables

## Data Availability

The data and materials of this research are available from the corresponding authors on reasonable request. The associated source codes are provided in https://github.com/wanglixuan1736/SGOC_scripts.git.
