# Supplementary material for: Integrative single-cell and bulk transcriptomes analyses reveals heterogeneity of serine-glycine-one-carbon metabolism with distinct prognoses and therapeutic vulnerabilities in HNSCC
Source: Int J Oral Sci. 2024 Jun 17;16:44. doi: 10.1038/s41368-024-00310-2 (PMC11183126; doi:10.1038/s41368-024-00310-2)
Supplement: Supplementary file 1 — Supplementary Figures [file 41368_2024_310_MOESM1_ESM.docx]

**Title:**

**Integrative single-cell and bulk transcriptomes analyses reveals heterogeneity of serine-glycine-one-carbon metabolism with distinct prognoses and therapeutic vulnerabilities in Head and Neck Squamous Cell Carcinoma**

**Authors and Affiliations:**

**Lixuan Wang^1,2,3†^, Rongchun Yang ^1,2,3†^, Yue Kong ^1,2,3^, Jing Zhou^1,2,3^, Yingyao Chen^1,2,3^, Rui Li^4^, Chuwen Chen^1,2,3^, Xinran Tang^4^,** **Xiaobing Chen^1,2,3^, Juan Xia^1,2,3^, Xijuan Chen^1,2,3^, Bin Cheng^1,2,3*^, Xianyue Ren^1,2,3*^**

^1^Hospital of Stomatology, Sun Yat-Sen University, Guangzhou, Guangdong, China.

^2^Guangdong Provincial Key Laboratory of Stomatology, Guangzhou, Guangdong, China.

^3^Guanghua School of Stomatology, Sun Yat-sen University, Guangzhou, Guangdong, China.

^4^Department of Radiation Oncology, Nanfang Hospital, Southern Medical University, Guangzhou, China.


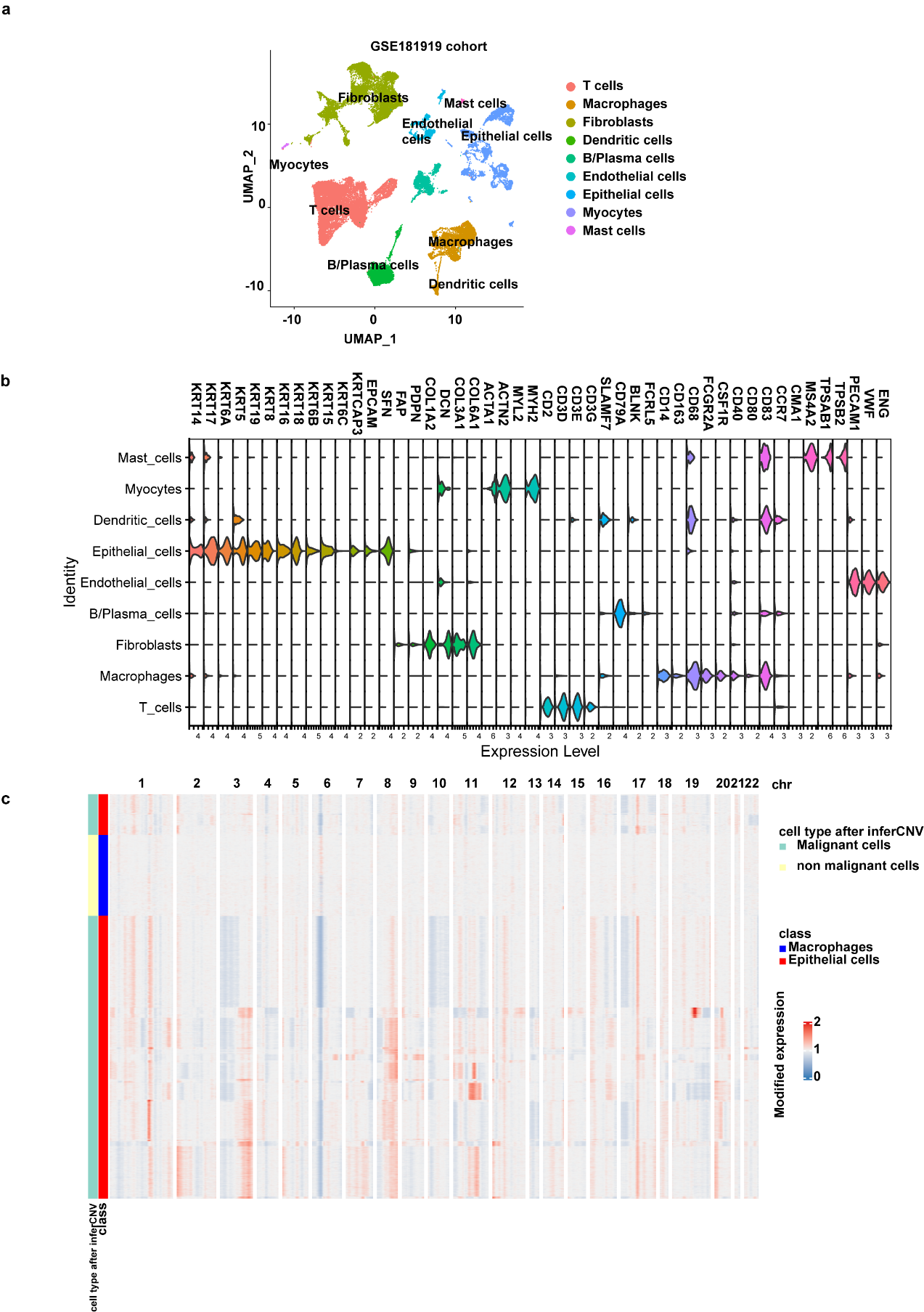


**Supplementary Figure 1. scRNA-seq profiling of HNSCC.** (a) UMAP plot of Cell types. (b) Makers of cell types. (c) CNV plot of epithelial cells.

**
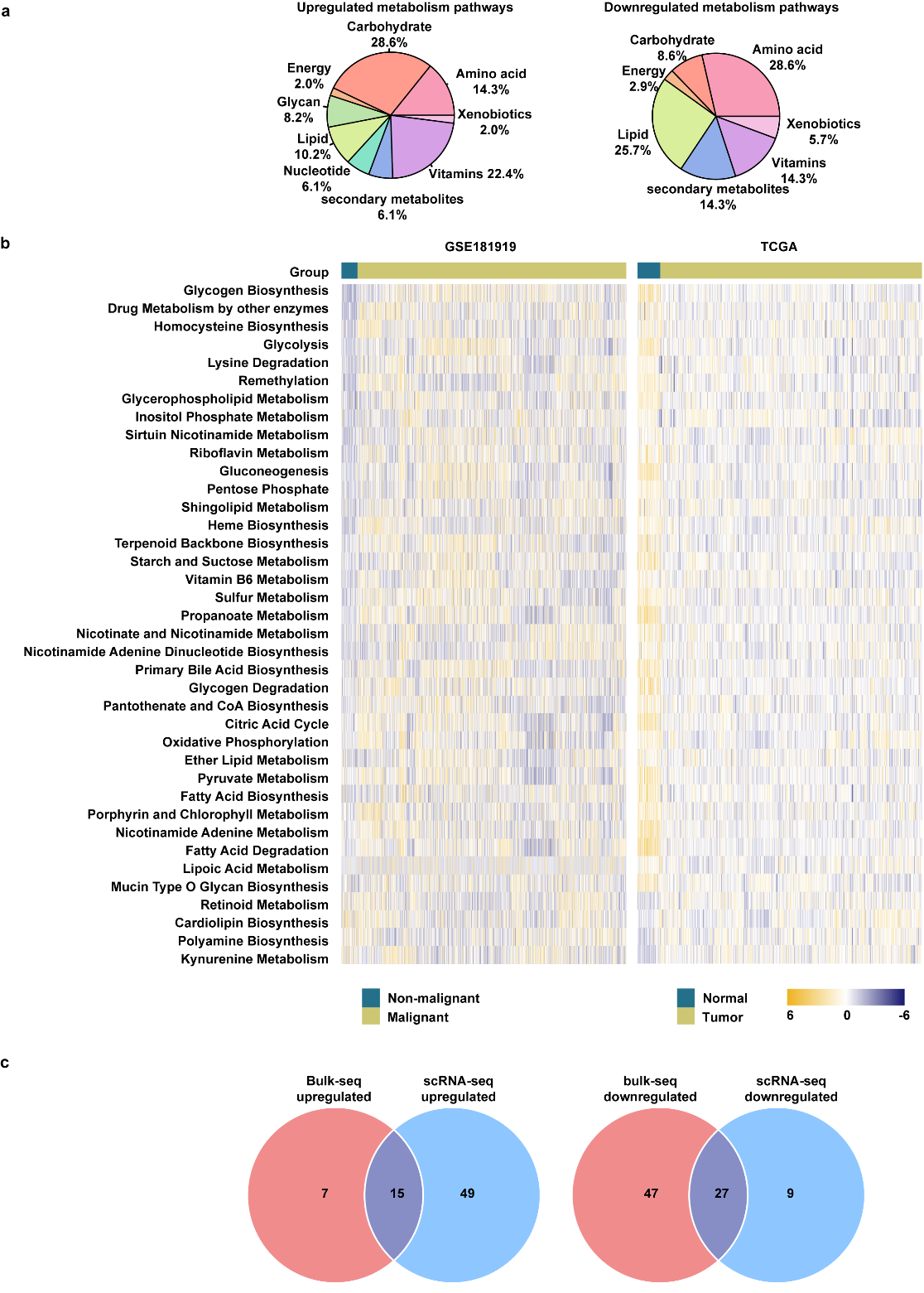
**

**Supplementary Figure 2. Rewiring of metabolic pathways in HNSCC.** (a) Proportions of altered metabolic pathways of 10 major metabolic types in malignant cells from scRNA-seq (GSE181919). (b) Dysregulated metabolic pathways in malignant cells (GSE181919) and bulk tumors (TCGA-HNSC) of HNSCC. (c) Venn diagram of differentially altered metabolic pathways in scRNA-seq (GSE181919) and bulk-seq (TCGA-HNSC).

**
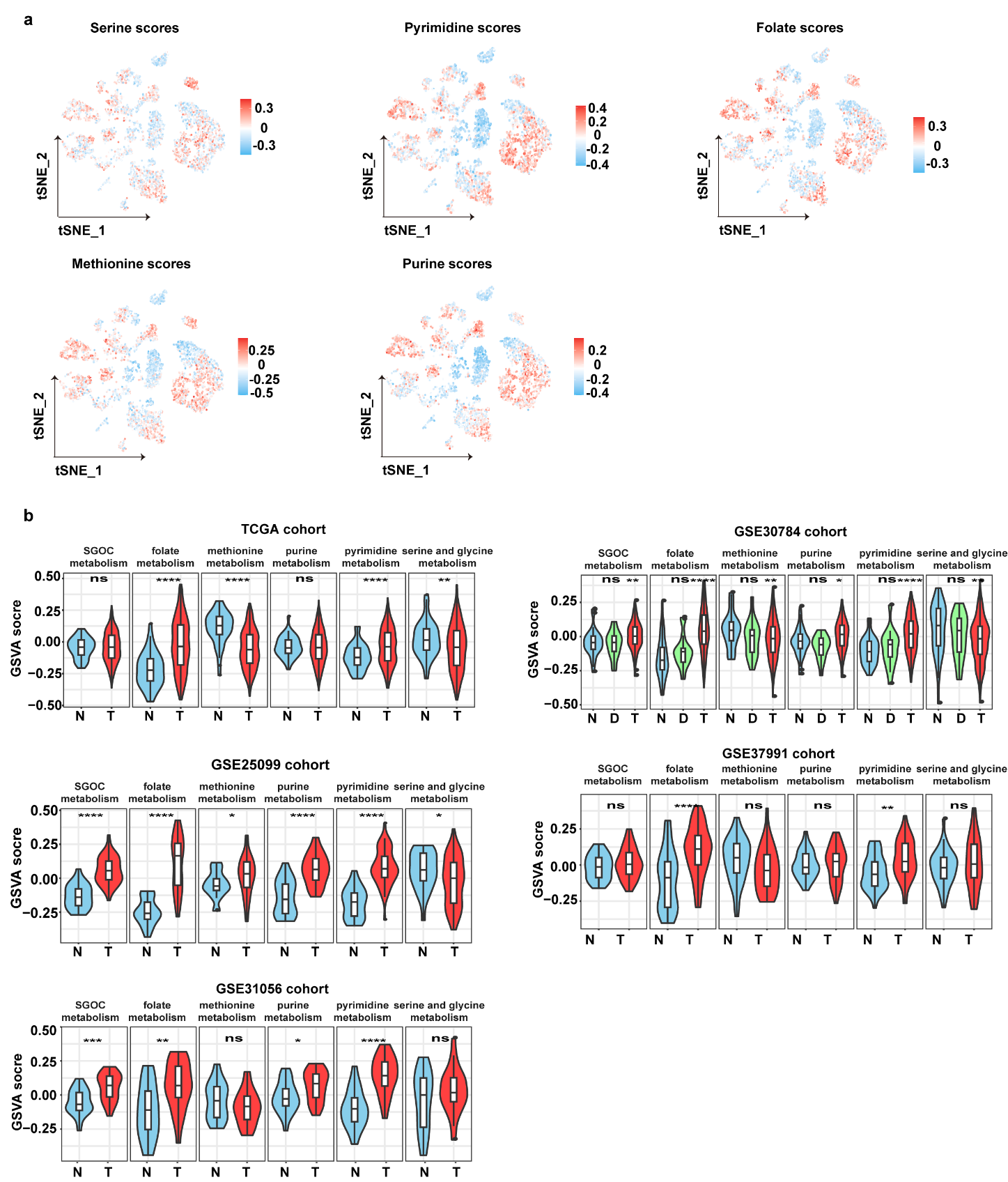
**

**Supplementary Figure 3. Alterations of SGOC metabolic pathways in HNSCC.** (a) UMAP plots of epithelial cells colored by SGOC cores from scRNA-seq (GSE181919). (b) Violin diagram shows the scores of SGOC metabolic pathways between normal and tumor bulk tissues.

**
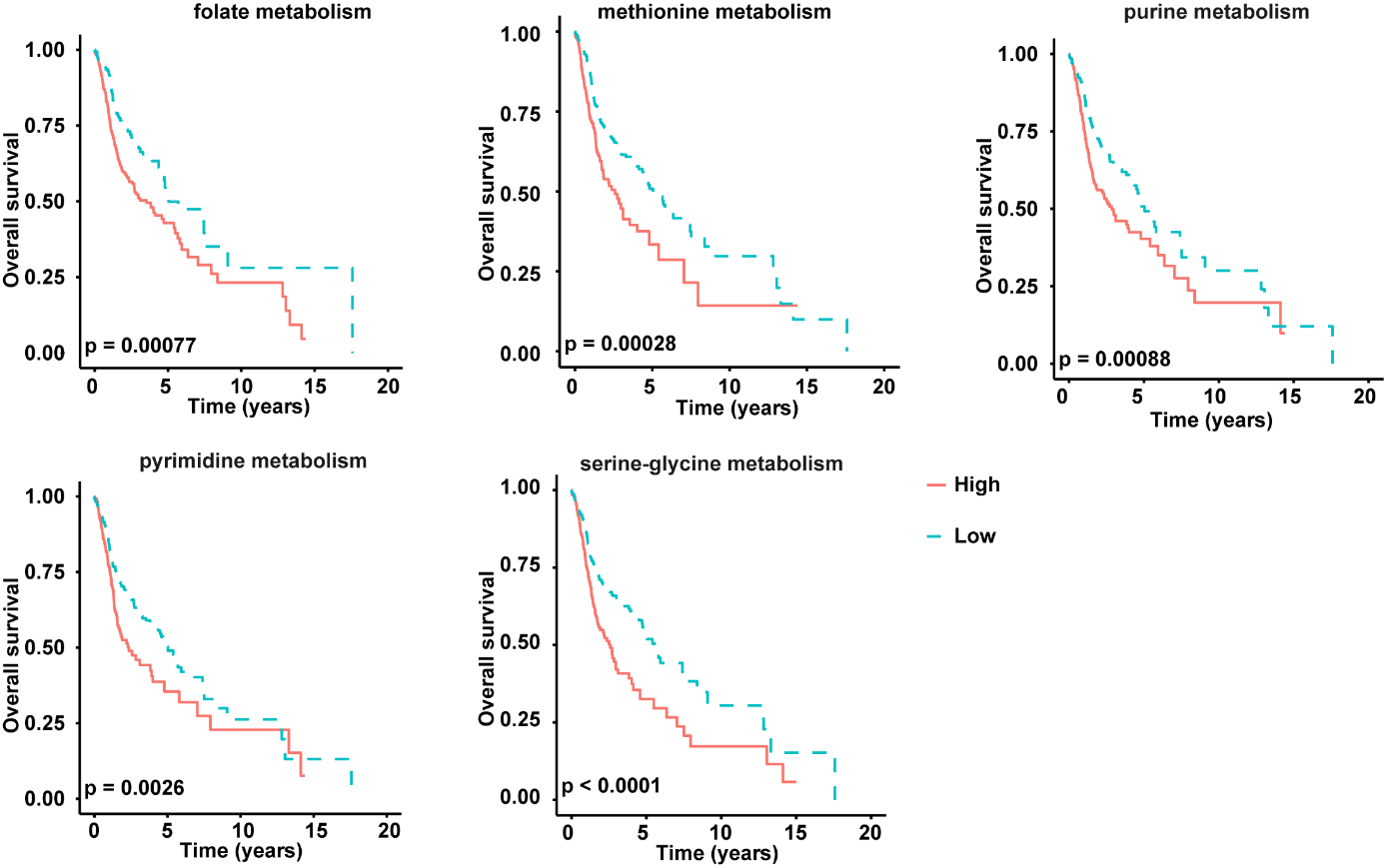
**

**Supplementary Figure 4. Kaplan–Meier curves of overall survival (OS) according to scores of SGOC metabolic pathways in TCGA-HNSC cohort.**

**
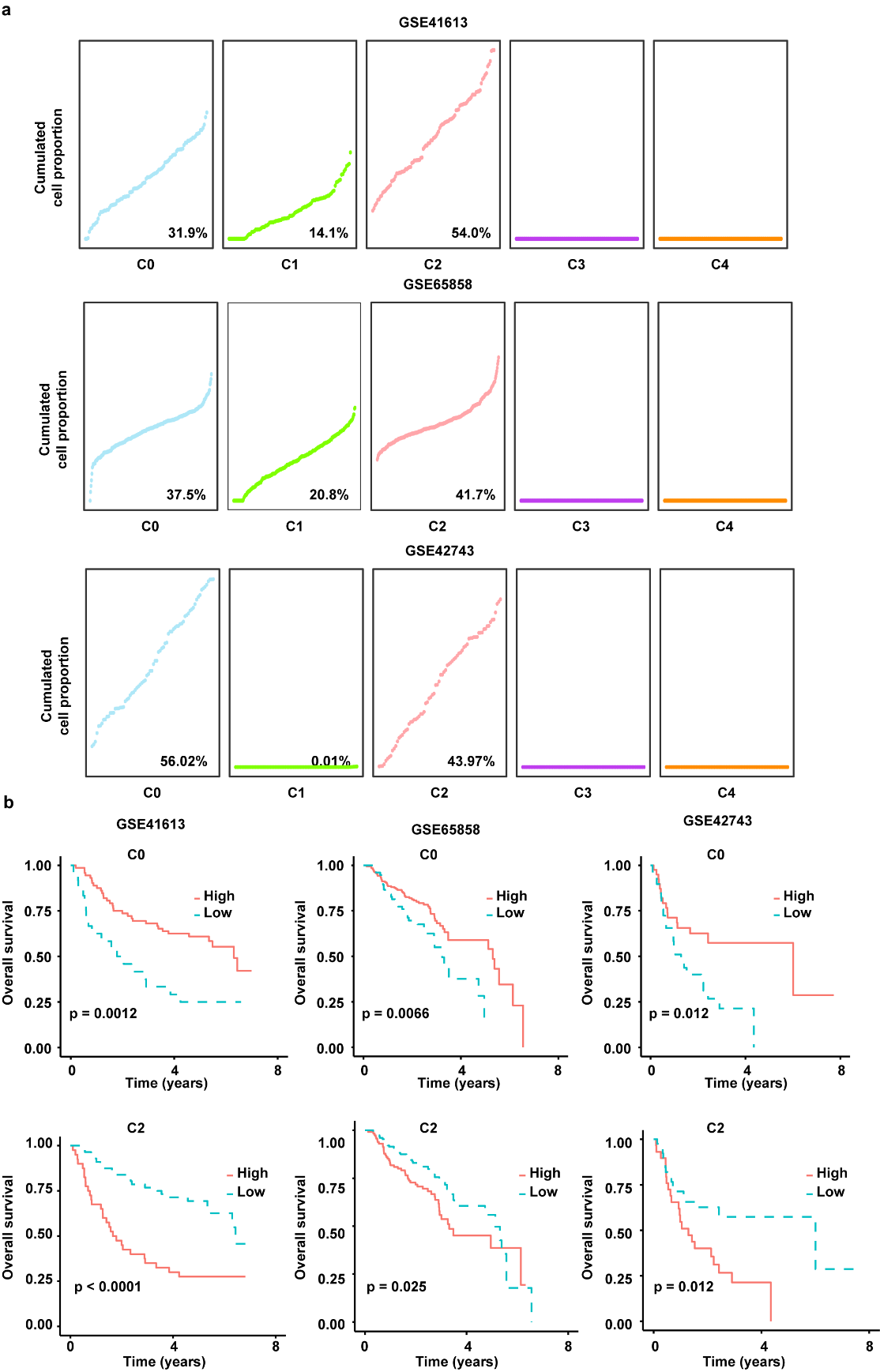
**

**Supplementary Figure 5:** (a) The cumulative cell proportion of the malignant cell clusters is shown in GSE41613, GSE65858 and GSE42743. (b) Kaplan–Meier curves of overall survival (OS) according to C0 and C2 clusters in GSE41613, GSE65858 and GSE42743.

**
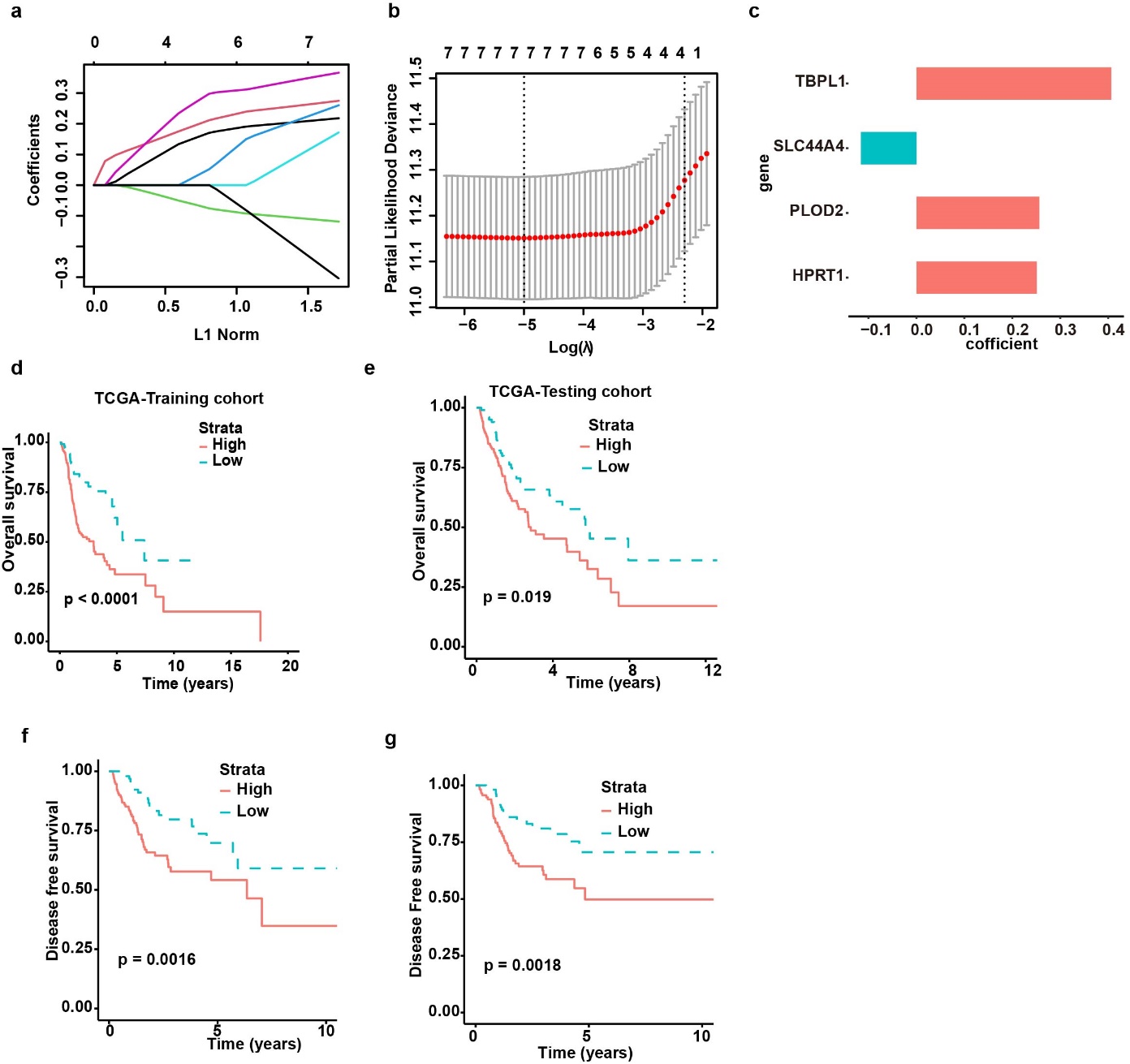
**

**Supplementary Figure 6. Construction of the SGOC-based prognosis signature.** (a) LASSO coefficient profiles of the 4-gene SGOC signature in training cohort of TGCA-HNSC. (b)Tuning parameter (λ) selection cross-validation error curve, the optimal log λ value is the left dotted line in the plot. (c) The coefficients of the 4 SGOC metabolic genes. (d-g) Kaplan–Meier curves for the OS and DFS of high-risk and low-risk groups in the training and testing cohorts of TCGA-HNSC.

**
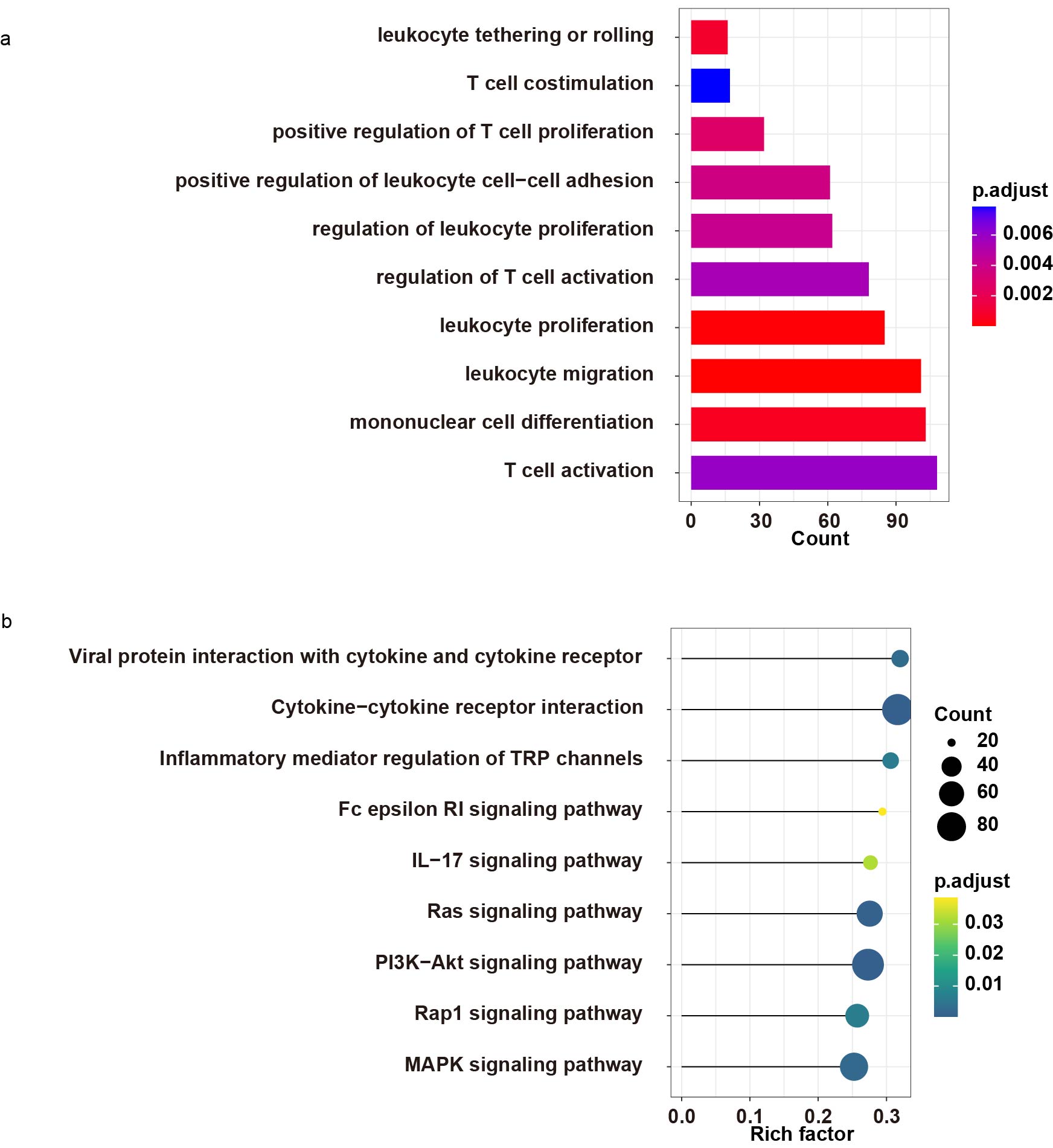
**

**Supplementary Figure 7. Pathway enrichment of differentially expressed genes between high- and low- risk patients in bulk tissues (TCGA-HNSC) determined by KEGG (a ) and GO analysis (b).**


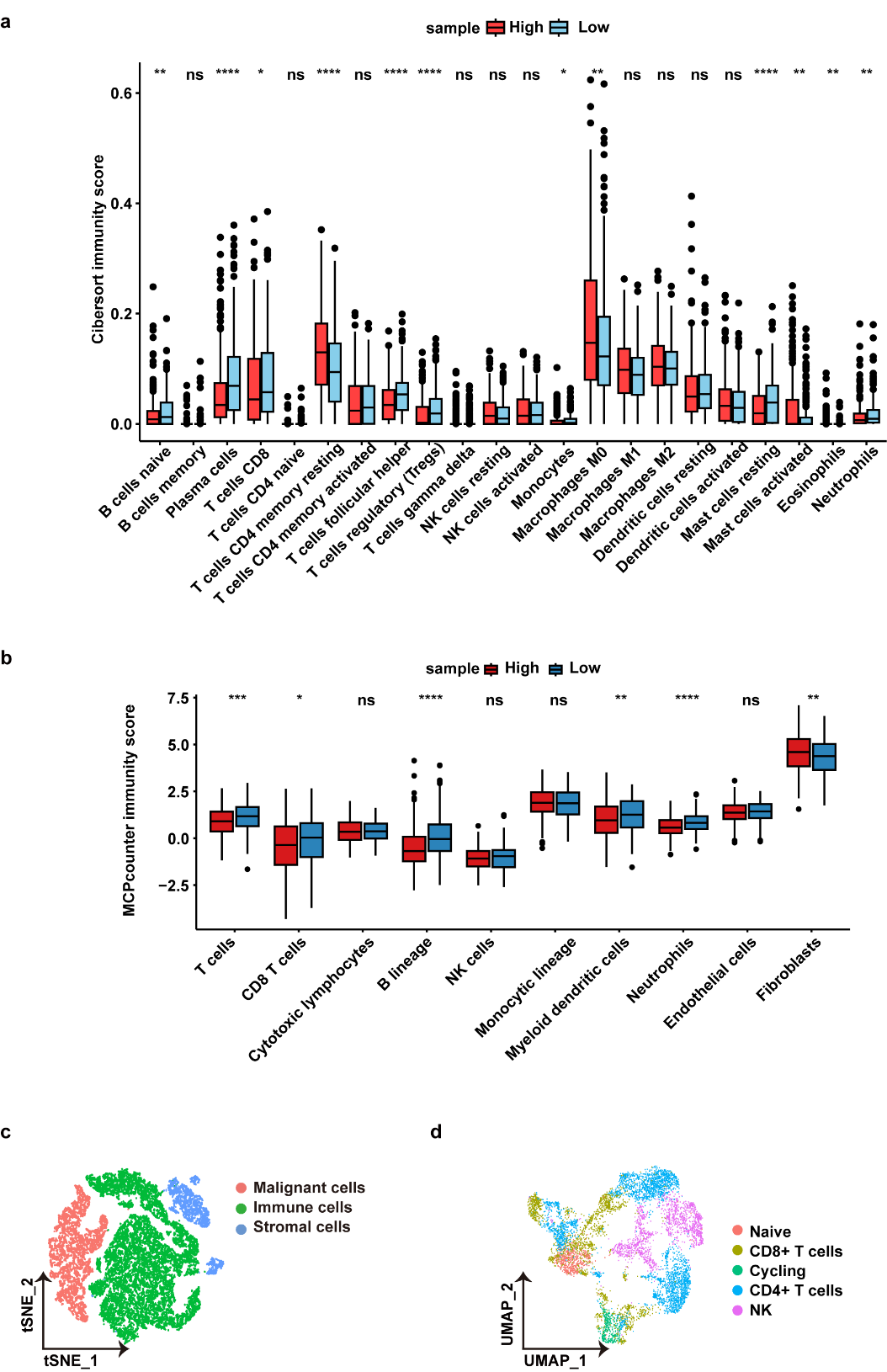


**Supplementary Figure 8. Immunity difference between SGOC-high, -low, and -median groups.** (a,b) The expressions of immune cells in the high- and low-risk groups of TCGA-HNSC cohort performed by “CIBERSORT”(a) and “MCPcounter” (c) tSNE plots of cells colored by cell type in scRNA-seq profiles. (d) UMAP plots of T cells colored by cell type in scRNA-seq profiles.


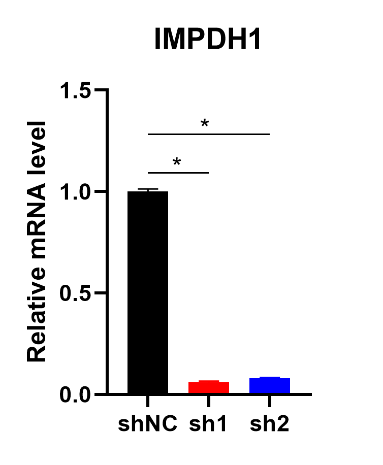


**Supplementary Figure 9.** **The transfection efficiencies of the shNC and shIMPDH1s**

**Plasmids were examined by qPCR. GAPDH was used as the endogenous control.**

**
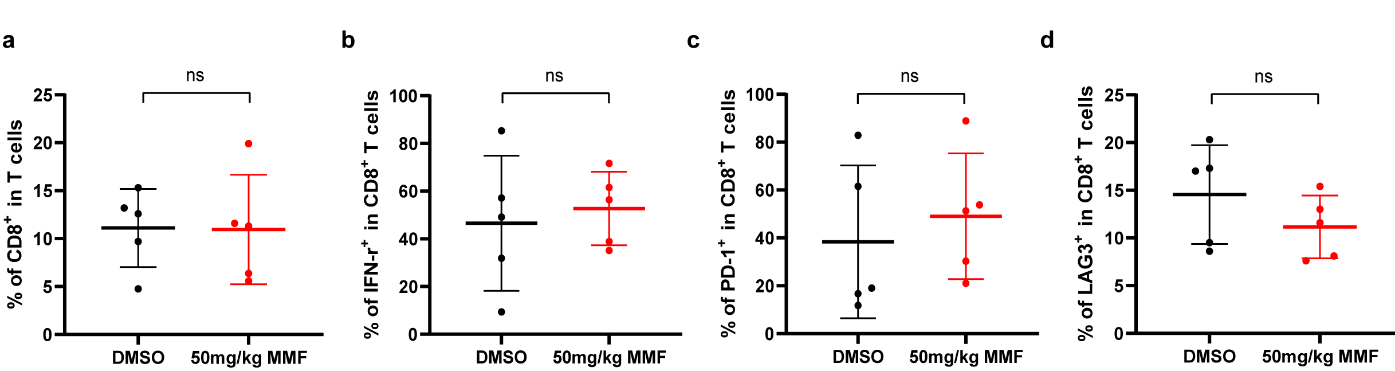
**

**Supplementary Figure 10.** **MMF had little effect on tumor-infiltrating CD8+ T cells.** (a) The proportion of infiltrated CD8+ T cells in the tumors of C3H mice. The proportion of IFN-γ^+^ (b), PD-1^+^ (c) and LAG3^+^ CD8+ T cells in the tumors.
